# The structure of *Shigella* virus Sf14 reveals the presence of two decoration proteins and two long tail fibers

**DOI:** 10.1038/s42003-025-07668-x

**Published:** 2025-02-12

**Authors:** Sundharraman Subramanian, Hailey R. Kerns, Samantha G. Braverman, Sarah M. Doore

**Affiliations:** 1 Department of Biochemistry and Molecular Biology, Michigan State University, East Lansing, MI 48824 USA; 2 Department of Microbiology and Cell Science, University of Florida, Gainesville, FL 32611 USA

**Keywords:** Bacteriophages, Cryoelectron microscopy

## Abstract

Bacteriophage Sf14 infects the human pathogen *Shigella flexneri*. A previous low-resolution structure suggested the presence of a decoration protein on its T = 9 icosahedral capsid. Here, we determined high-resolution structures of the Sf14 capsid and neck, along with a moderate-resolution structure of the whole Sf14 tail and baseplate. These structures indicate the capsid has not one, but two different types of decoration proteins: a trimeric β-tulip lattice that covers the entire capsid and a set of Hoc-like proteins that bind preferentially to hexamers at the quasi-3-fold axes of symmetry. The neck also contains two sets of whiskers oriented in opposite directions, and the tail has two types of long tail fibers which may bind different receptors. Based on homology and phylogenetic analysis, Sf14 may be the product of multiple horizontal gene transfer events. The structures presented here can be used to investigate further hypotheses of phage structure-function relationships and structural diversity.

## Abstract (web summary)

Structures of Shigella bacteriophage Sf14 show similarity with several features from diverse bacteriophages that, when combined, produce a T = 9 icosahedral capsid coated in decoration proteins and a contractile tail with two tail fibers.

## Introduction

Bacteriophages, also known as phages, are viruses that infect bacteria. They are considered the most abundant biological entities on Earth, with an estimated 10^31^ particles at any given time^1^. New phage genotypes are continually being discovered through metagenomic and phage-hunting efforts, suggesting bacteriophages are extremely diverse^2,3^. Although these studies can indicate which phages are where and what their genomes encode, investigating their specific characteristics is more complicated. In addition to genome content and organization, one way to classify phages is through morphology. Most previously described phages use icosahedral capsids to protect their genomes from the environment. These capsids can be further classified based on their symmetry, which is represented by triangulation (or *T*) number. Many phages also have a head-tail morphology, meaning these capsids are bound to tail structures.

Numerous phages—including the model systems λ, P22, and T7—package their genomes into capsids with *T* = 7 symmetry, while phages with larger genomes typically use capsids with *T* = 13 symmetry (as reviewed in ref. ^4^). Until recently, only two phages had been described with intermediate *T* = 9 symmetry^5,6^, but this number has increased over the past few years^7–10^. This may have been due to a previously limited collection of phages and phage-host pairs or to inherent unfavorable properties of *T* = 9 capsids^4,11^. The common presence of decoration proteins^10^, which may serve to stabilize capsids, could support the hypothesis that *T* = 9 capsids have lower persistence in the environment.

With more phages being isolated on a greater variety of host organisms, we are observing an increase in both structural and genetic diversity^3^, including an increase in *T* = 9 structures. While these *T* = 9 phages all appear to use decoration proteins or minor capsid proteins, each binding mechanism and organization thus far appears to be unique^10,12^. We previously reported a low-resolution structure of Sf14, a virus that infects the enteric bacterial pathogen *Shigella flexneri*^13^. This initial structure indicated a decoration protein that binds to the center of each hexamer at the quasi-threefold axis of symmetry, but other features were unclear. Here, we report multiple high-resolution structures of the full Sf14 particle, including symmetric and asymmetric reconstructions of the virion capsid, empty capsid, portal region, tail, and baseplate.

The capsid structures indicate that Sf14 utilizes two decoration proteins on its capsid: a minor capsid protein gp33 and a larger decoration protein gp20. The latter is structurally similar to T4 hoc and binds at the center only of hexamers surrounding the five-fold axes of symmetry^14^. In addition to the virion capsid, we determined the structure of spontaneous empty capsids, which have a mixture of non-attached or attached tails, the latter of which are contracted or non-contracted, but contain no genome. These particles are prevalent in typical Sf14 preparations and were hypothesized to be destabilized capsids lacking at least one decoration protein. However, these proteins are still present in the empty capsids and the etiology of these particles remains elusive.

Other structures resolved here indicate that Sf14 particles have two sets of whiskers, two types of tail fibers, a Mu-like baseplate, and a central tail needle bound to a terminal dome-shaped cap. The tail structure is overall similar to many Mu-like viruses^15^, with its use of two tail fibers having implications for host range and recognition^16^. Although it shares many similarities with Mu, Sf14 also has some specific structural properties that warrant further investigation, including longer tail fibers and a cap on the tail needle.

## Results and discussion

### Structural features of the Sf14 virion and capsid

**Fig. 1:**
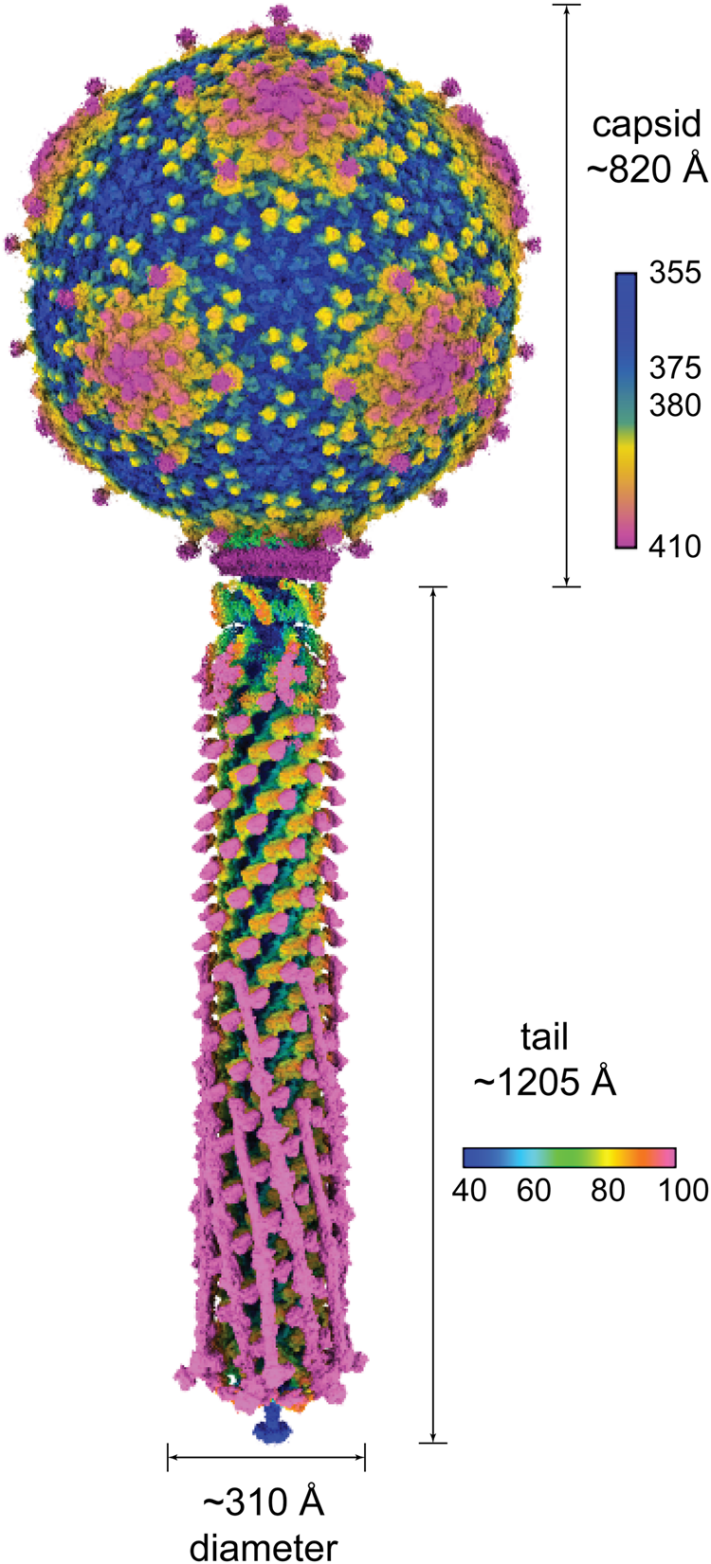
A composite view of the Sf14 virion, with an asymmetric map of the capsid and C6 reconstruction of the tail. Dimensions are reported in Å. Maps are colored according to radial distance in Å from the center of the capsid or from the center of the tail tube. Note the different scales of the color keys between the capsid and tail.

Sf14 virions have a spherical icosahedral capsid of 820 Å in diameter, which is connected to a long, contractile tail that is approximately 1205 Å long and an average of 235 Å in diameter, expanding to 315 Å at the baseplate (Fig. 1). In addition to virions, typical Sf14 preparations include a large proportion of empty capsids: a representative micrograph is shown in Supplemental Fig. S1. These empty particles do not always have a contracted tail and maybe without a tail completely, though the capsid appears to be mature. They may have lost their genomes due to instability rather than spontaneous ejection or delivery into the host cell. Whether this instability originates at the capsid, neck, baseplate, or elsewhere is unclear. It is also unclear whether this is a biological phenomenon or an artifact of preparation prior to imaging. To determine the structure of virions specifically, only particles containing the genome were used for reconstruction.

Like all *T* = 9 capsids, the virion has two types of hexamers that are present at the 3-fold and quasi-3-fold axes of symmetry. Also consistent with many *T* = 9 phages, the Sf14 capsid is rough due to the presence of decoration proteins. However, while many other *T* = 9 phages have one reported decoration protein, Sf14 appears to have two: one that is present as trimers surrounding each hexamer and a second that is at the center of each quasi 3-fold hexamer. Density for this second decoration protein was visualized on a previously published ~15 Å map^13^ and can also be seen as protrusions in the 4.5 Å asymmetric reconstruction here in Fig. 1.

**Fig. 2:**
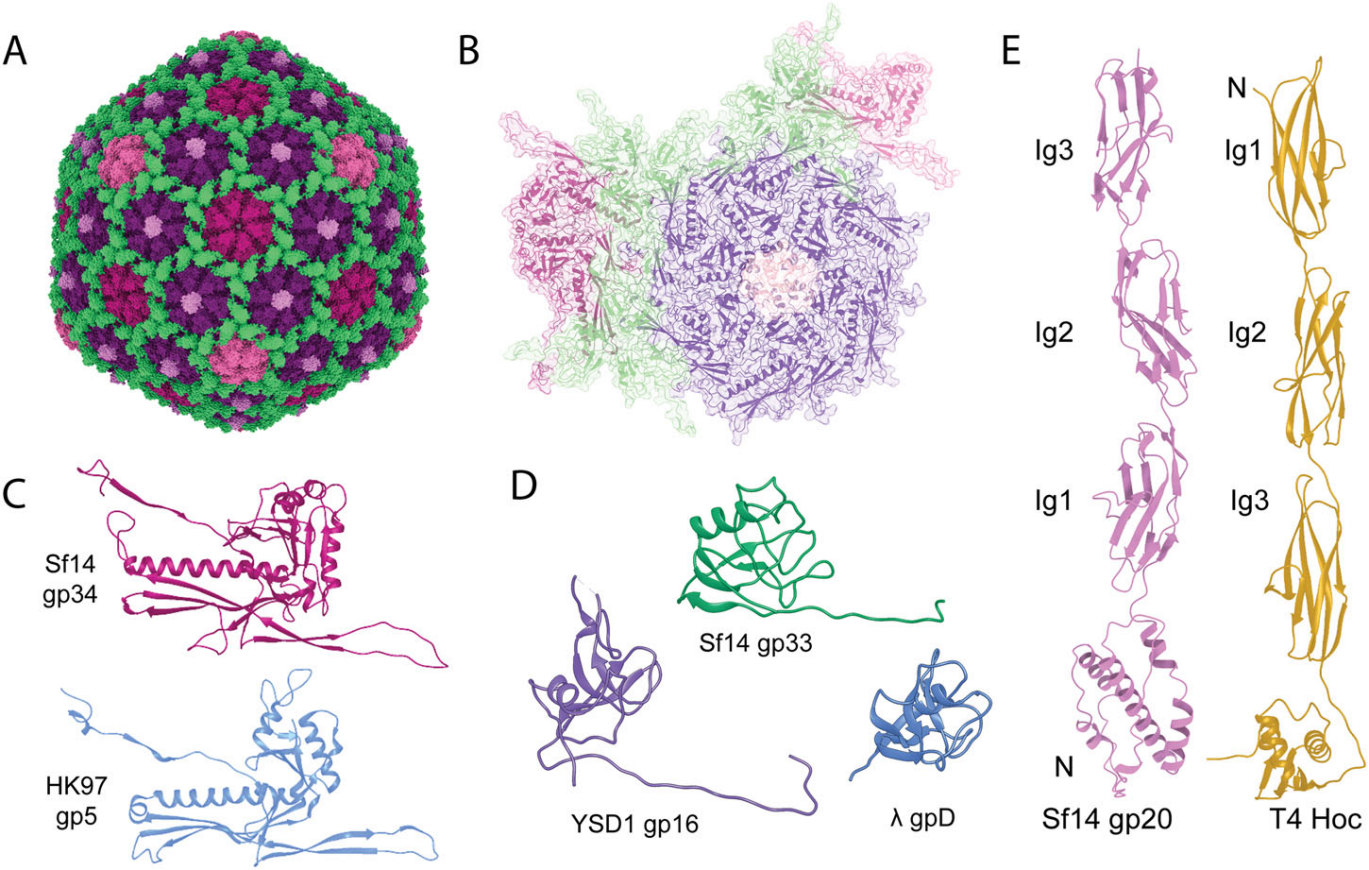
The capsid and decoration proteins of the Sf14 virion. Models of the (**A**) full virion and (**B**) virion asymmetric unit. The gp34 capsid proteins of each capsomere are colored according to location: purple at the quasi 3-fold axis of symmetry, magenta at the true 3-fold, and rose pink at the 5-fold. The decoration protein gp33 is green, with the decoration protein gp20 in light pink. A comparison of (**C**) Sf14 capsid protein gp34 with HK97 capsid protein gp5; and Sf14 decoration proteins (**D**) gp33 compared to other beta-tulips, and (**E**) gp20 compared to T4 hoc. Note: the gp20 model shown here is predicted by AlphaFold, and the capsid-binding domain is not reflective of the density of the capsid. The AlphaFold model was left unchanged in the figure to emphasize this is a prediction.

The capsid structure was then resolved to a higher resolution of 3.2 Å via symmetric reconstruction (Fig. 2A). This reconstruction has a maximum diameter of 801.6 Å when measuring between the surfaces of opposing pentamers and 758.5 Å between internal surfaces, with pentamers being approximately 43 Å thick. This diameter is smaller than the asymmetric reconstruction due to the absence of the protruding decoration protein, which was averaged out during data processing. Next, protein structures were predicted and modeled into the density of the asymmetric subunit (Fig. 2B) using ModelAngelo^17^. With these methods, we determined the identity of both decoration proteins: gp33 and gp20. Each asymmetric subunit contains nine chains of capsid protein gp34, three trimers of decoration protein gp33, and one monomer of decoration protein gp20. Due to disparities in gene product nomenclature, all structural gene products with established numbers and the new GenBank locus tags are presented in Table 1.

**Table 1:** A list of Sf14 gene products, including their current locus tag in GenBank, the protein encoded, and whether it was detected via mass spectrometry analysis of particles

Gene Product	Current Locus Tag	Protein	Detected via MS
gp20	FDI99_gp20	Decoration (hoc-like)	Yes
gp29	FDI99_gp128	Portal	Yes
gp30	FDI99_gp127	Whisker	Yes
gp31	FDI99_gp126	Whisker	Yes
gp33	FDI99_gp124	Decoration (ß-tulip)	Yes
gp34	FDI99_gp123	Capsid	Yes
gp35	FDI99_gp122	Head-to-tail connector	Yes
gp37	FDI99_gp120	Adapter	Yes
gp38	FDI99_gp119	Collar	Yes
gp39	FDI99_gp118	Tail sheath	Yes
gp40	FDI99_gp117	Tail tube (rv5 Orf152-like)	Yes
gp41	FDI99_gp116	Tape measure chaperone?	No
gp42	FDI99_gp115	Tail assembly chaperone?	Yes
gp43	FDI99_gp114	Tape measure protein	Yes
gp44	FDI99_gp113	Bottom of tail tube, Afp7 homolog	Yes
gp45	FDI99_gp112	Genome circularization protein?	Yes
gp46	FDI99_gp111	Central baseplate hub, Mu gp44 homolog	Yes
gp47	FDI99_gp110	Central needle	Yes
gp48	FDI99_gp109	Wedge subunit	Yes
gp49	FDI99_gp108	Wedge subunit	Yes
gp50	FDI99_gp107	Wedge subunit	Yes
gp51	FDI99_gp106	Homolog of T4 gp11?	No
gp52	FDI99_gp105	Long tail fiber	Yes
gp53	FDI99_gp104	Longer tail fiber	Yes
gp55	FDI99_gp102	Holin or lysozyme	Yes

The major capsid protein gp34 has a traditional HK97 fold, with an E-loop and spine helix like the HK97 major capsid protein (PDB 1OHG, Fig. 2C). The primary differences are in the number of β-sheets and the presence of insertion loops. The A-domain also contains two short α-helices at the apical region, which mediates interactions with other A-domains within a pentameric or hexameric multimer. The center of the Sf14 capsomer is packed with α-helices and lined with negatively-charged Asp residues, while HK97 capsomers are filled with loops and contain Glu and Arg residues. Additional comparisons between Sf14 gp34 and structurally similar capsid proteins are presented in Supplemental Text and Supplemental Fig. S2.

### The two types of Sf14 decoration proteins suggest different roles

The bumpy appearance of Sf14 capsids can be attributed to the aforementioned decoration proteins, identified here as gp33 and gp20. The gp33 monomer is largely composed of loops, with five β-sheets, one α-helix, and two helical turns (Fig. 2D). Overall, the gp33 structure and organization resembles the traditional β-tulip motif seen in gpD (*λ*), gp87 (P74-26), and gp8.5 (φ29) (as reviewed in ref. ^18^). Based on a DALI search^19^, the Sf14 gp33 monomer is most similar to phage YSD1’s gp16. A comparison showing the β-tulip YSD1 gp16 (*Z*-score 7.7) and λ gpD (*Z*-score 7.2) proteins is also shown in Fig. 2D.

The identity and structure of the second decoration protein have been unclear since^13^ but was hypothesized to be gp20. Based on the AlphaFold prediction, gp20 closely resembles that of phage T4 Hoc (highly immunogenic outer capsid) protein (Fig. 2E). The structure and function of Hoc were recently reported in ref. ^14^ but was historically elusive due to multiple binding orientations on the capsid. Hoc forms fiber-like extensions away from the capsid that are hypothesized to sense environmental conditions and facilitate particle aggregation. Hoc preferentially occupies one of two preferred orientations out of the six possible orientations, which facilitated the resolution of its capsid-binding domain. Beyond the capsid binding domain, T4 Hoc has three immunoglobulin-like (Ig-like) domains that extend away from the capsid surface. Sf14 gp20 is also predicted to contain three Ig-like domains which may serve a similar function. The Sf14 cryoEM map density suggests gp20 occupies only one orientation, as we were readily able to determine its capsid binding domain structure here.

Sf14 gp20 is homologous to many other immunoglobulin (Ig) domain-containing proteins in both phages and multiple bacterial species. While Ig-like proteins are variable in sequence and structure, they tend to have a Greek-key β-sandwich core structure^20^. Based on InterproScan, portions of gp20 and its homolog vB_EcoM_AYO145A gp41 were identified to be immunoglobulin superfamily carcinoembryonic antigen-related (IgSF-CEA) proteins, which mediate intercellular adhesion or interactions with the extracellular matrix^21^. Similarly, TreeGrafter^22^ annotated gp20 and homologs vB_EcoM_AYO145A gp41 and KPS64 gp94 as cell surface proteins involved in heterophilic cell-cell adhesion via plasma membrane cell adhesion molecules. The vB_EcoM_AYO145A decoration protein also has filamin repeat-like domains, which are actin cross-linking proteins. Combined, this suggests gp20 could be aggregating Sf14 particles together like T4 Hoc, to components of the environment, or to host cells.

**Fig. 3:**
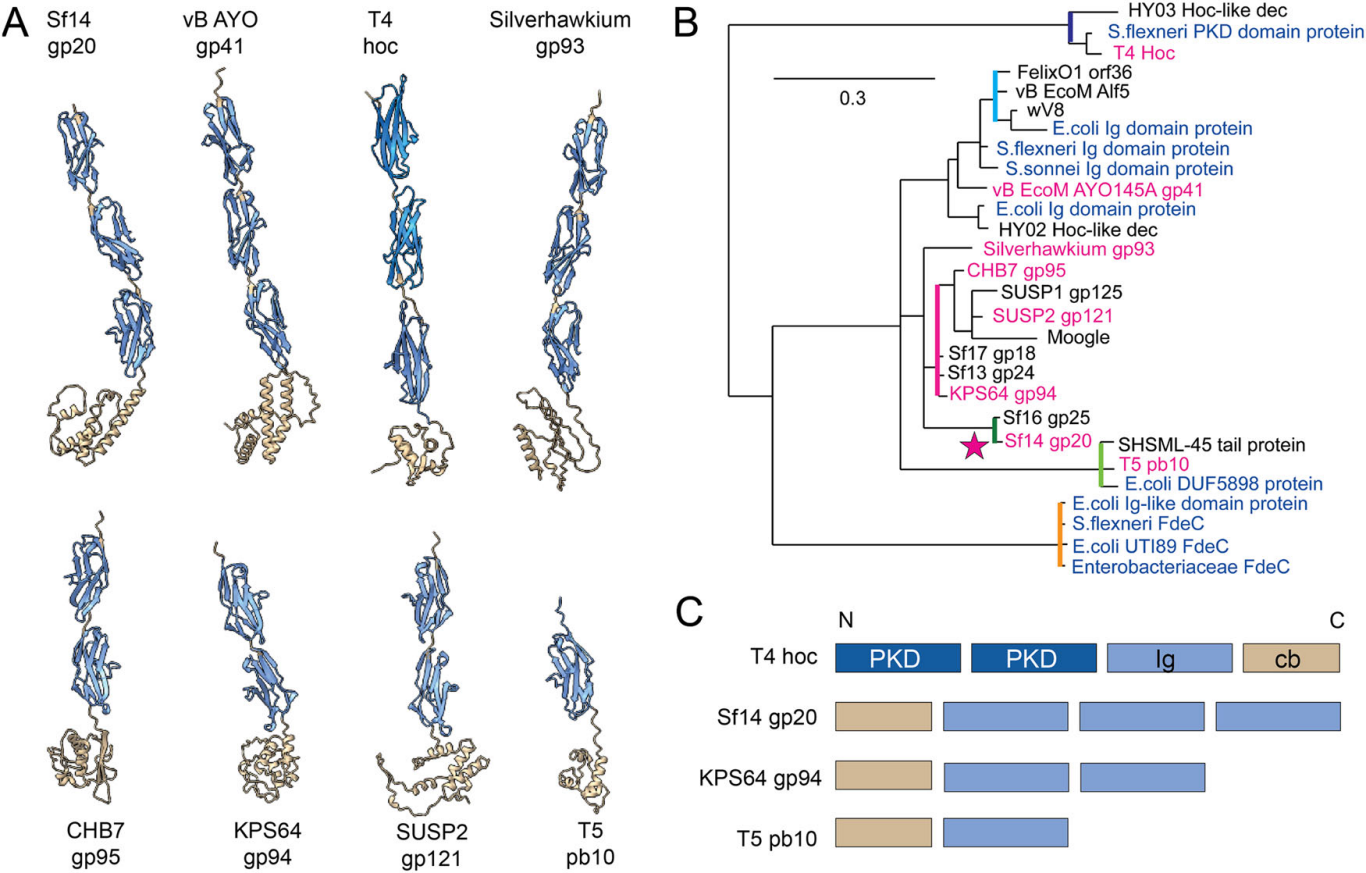
Evolutionary context of the Sf14 gp20 decoration protein. **A** Structures of Sf14 gp20 alongside its homologs. Cornflower (lighter blue) shows Ig-like domains while dodger blue (intense blue) shows PKD domains (Ig-like domains thought to aid in cell-to-cell adhesion). Beige is for capsid-binding (cb) domains. **B** A phylogeny based on protein sequence, colored by clade: dark blue, T4; light blue FelixO1; pink, CHB7, SUSP2, KPS64; dark green, Sf14; light green, T5; orange, bacterial Ig-like domain-containing proteins. Phage Sf14 is also denoted by a pink star. All bacterial homologs are in blue text, and all phage homologs shown in A are indicated by pink text. **C** Domains of T4, Mooglevirus, and T5 homologs, colored according to the representations in A. Interestingly, all of them have their N-terminus in the capsid binding domain except T4 hoc.

Decoration proteins with Ig-like domains appear in numerous phages, but the number of these domains is variable. In terms of model phages, T4 Hoc contains three Ig-like domains while T5 pb10 contains one (Fig. 3A^14,23^;). The gp20 homologs of other related viruses, including Silverhawkium gp93 and vB_AYO145A gp41, also have three. Interestingly, CHB7 gp95, KPS64 gp94, and SUSP1 gp121 only contain two Ig-like domains. This was also observed for *Shigella* phages Sf13 and Sf17. The functional and evolutionary difference between the number of domains is unclear, especially since there is no significant difference in host range between these groups, or even a difference in geographic location between Silverhawkium, CHB7, and KPS64^24^. Since many bacteria encode proteins for adhesion, it is possible additional domains were added through recombination of the phage genome with these host genes. Conversely, the loss of Ig-like domains could have occurred. A comparison between Sf14 gp20 and CHB7 gp95 indicates multiple premature stop codons in the latter, though there are also several insertions and deletions that could be explained by either hypothesis (see Supplemental Alignment). The phylogeny shown in Fig. 3B was constructed from homologs of Sf14 gp20, CHB7 gp95, T4 Hoc, and T5 pb10 from multiple different phages. It does not indicate the clustering of three Ig-domain phages based on protein sequence. An additional Ig-like domain may have arisen independently in several lineages.

**Fig. 4:**
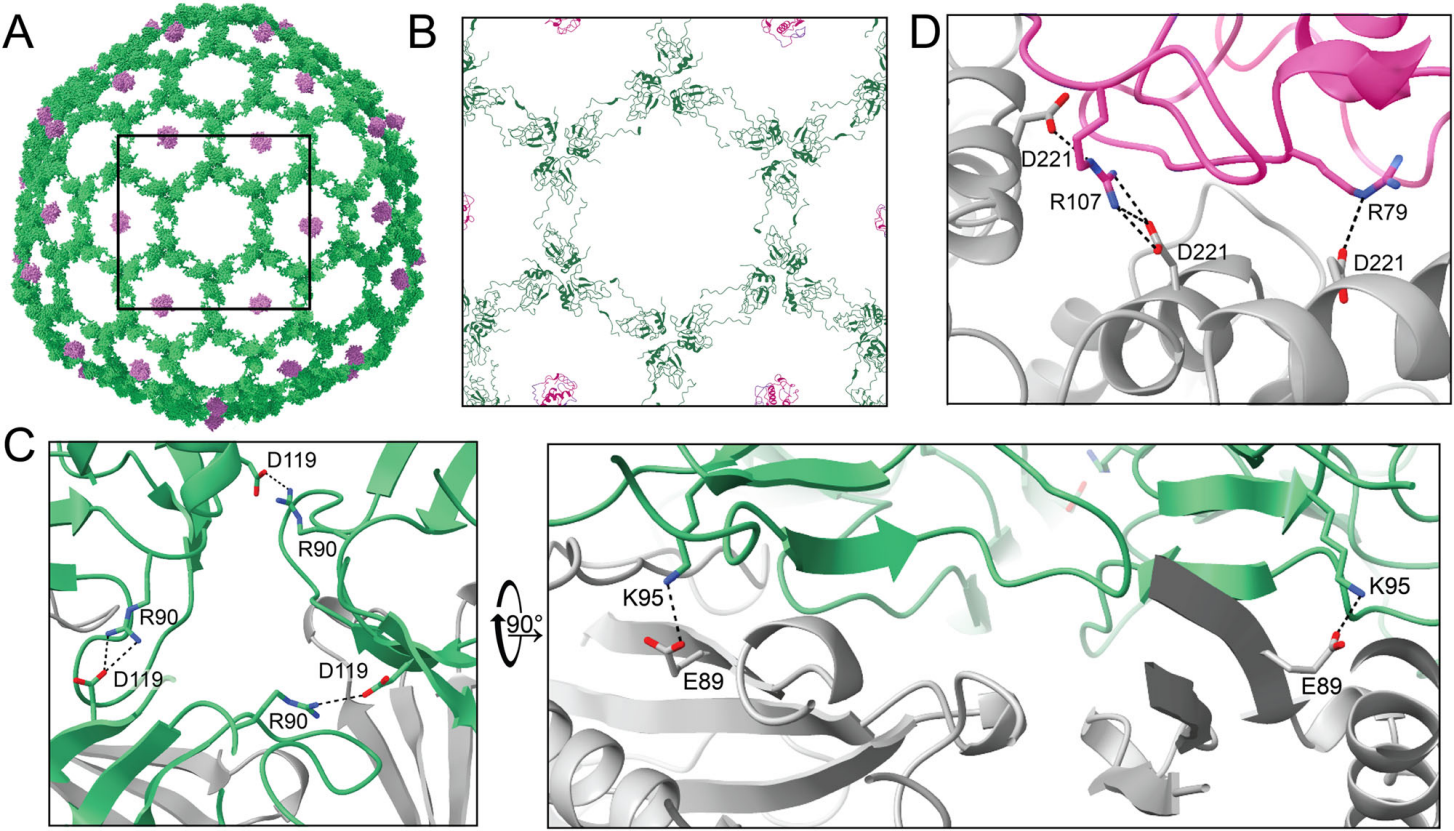
Sf14 decoration proteins on the virion and their mechanism of binding. **A** Model of the virion with only decoration proteins is shown, with gp33 shown in green and gp20 depicted in pink. **B** A close-up of the box shown in (**A**) represented in ribbon form, emphasizing the interacting N-arms between trimers of gp33. **C** Specific residues forming salt bridges between green gp33 proteins at the trimer interface (left) and between each gp33 and gray gp34 subunit. **D** Interactions between pink gp20 and gray gp34 at the center of pentamer-proximal hexamers.

A global view of both decoration proteins is shown in Fig. 4A, with trimers of decoration protein gp33 forming a lattice that covers the capsid and gp20 occupying only the pentamer-proximal hexamers at the quasi 3-fold axis of symmetry. The gp33 lattice—or chain mail—could be consistent with the hypothesis that decoration proteins are involved in capsid stability^18^. The lattice is formed by the N-terminal arm of each gp33 monomer interacting with its neighboring monomer (Fig. 4B). Within a gp33 trimer, the trimeric interface is stabilized through salt bridges between Asp and Arg on neighboring subunits (Fig. 4C left). The trimer is then anchored to the capsid protein gp34 by additional salt bridges involving gp33 Lys and gp34 Glu (Fig. 4C right).

The decoration protein gp20 binds the capsid as a monomer, with the capsid binding domain contained in the N-terminal 118 amino acids, folded into a largely helical bundle. The core of this domain is hydrophobic, while the external surface is enriched in charged and polar residues. This domain is also anchored to the gp34 hexamer by salt bridges, with positively-charged gp20 Arg residues 79 and 107 interacting with three of the six gp34 monomers at Asp 221 (Fig. 4D left). The specificity of gp20 for quasi 3-fold hexamers could be due to differences at one of the three gp34 monomers. The two hexamers were overlaid to compare the orientation of the critical residues in gp34. In the true 3-fold hexamers, two of the three Asp 221 residues are shifted away from both gp20 Arg 79 and Arg 107, with the Arg 79 interacting side-chain oriented away from gp20 (Fig. 4D right, arrow). This results in a single bond between gp20 and gp34 rather than the multiple shown in Fig. 4C between gp33 and gp34.

We hypothesized that the large proportion of empty capsids could be due to a loss of one or both decoration proteins. However, after resolving a separate structure based on empty particles alone, we did not see observable differences in the capsid composition or conformation (Supplemental Text and Supplemental Fig. S3). Additional studies are needed to investigate both the structure and biological relevance of these particles.

### The Sf14 neck contains a dodecameric portal protein complex and two sets of whiskers

**Fig. 5:**
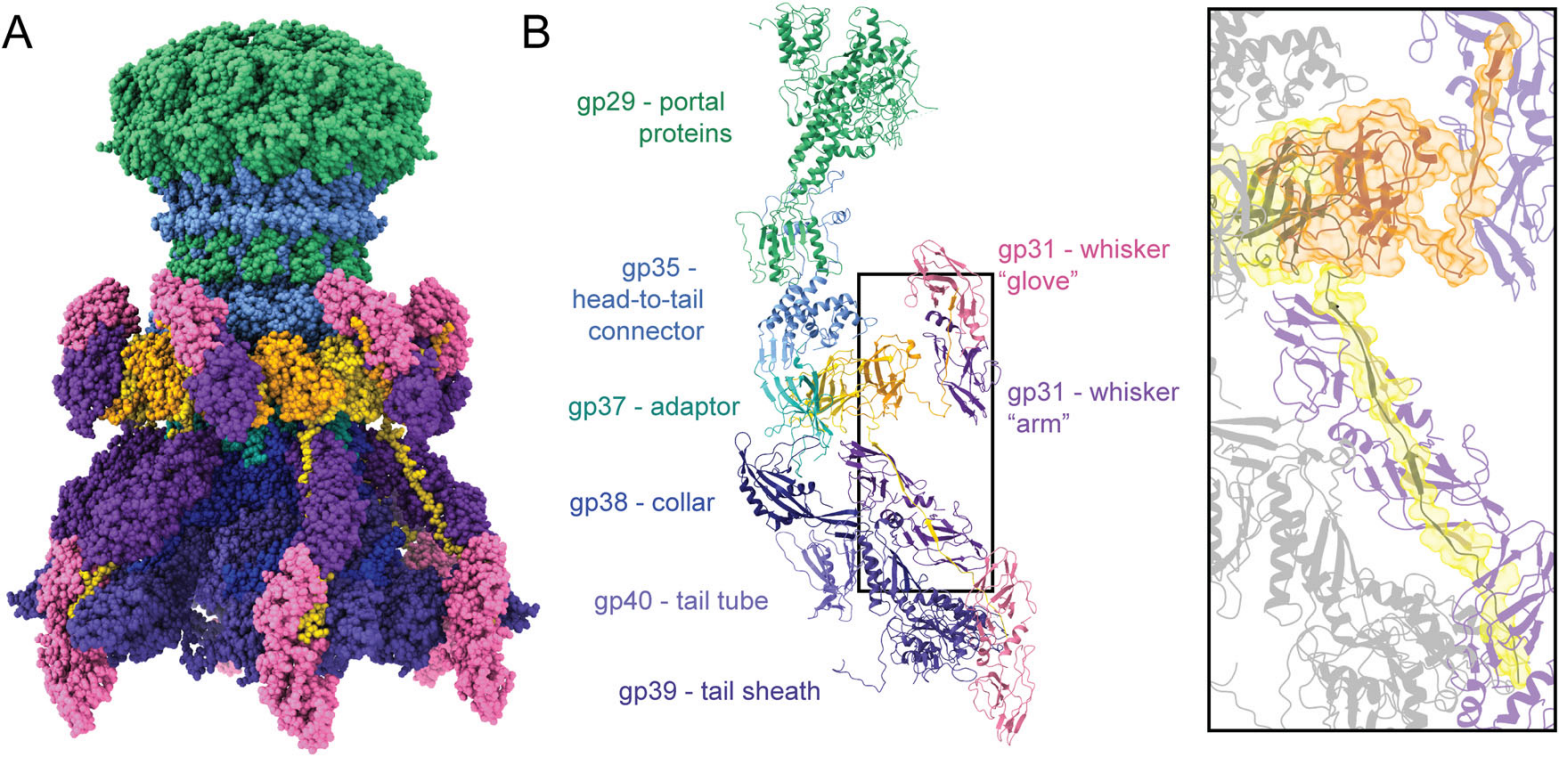
The Sf14 neck region, including the portal and two whiskers. **A** Space-fill model using C6 symmetry on (**B**) the asymmetric subunit ribbon diagram with proteins labeled. The zoom box shows two subunits of gp30 (outlined in orange and yellow) threading up and down into each whisker (purple).

We determined the structure of the Sf14 neck or portal region next, with the map resolved to 3.4 Å resolution (Fig. 5A). As the connector between capsid and tail, portal regions are often complex and contain numerous proteins. Here we used ModelAngelo to predict and model the individual protein chains in this region (Fig. 5B). In addition to the portal protein gp29, two sets of whiskers are seen between the capsid and tail. One set of whiskers points “up” toward the capsid, while the other set points “down” towards the tail. Each set of whiskers is made of four protein chains: two of gp30 and two of gp31, though one chain of gp30 threads through both up and down whiskers (Fig. 5B inset). The connecting copy of gp30 may be involved in maintaining the up/down conformation, though how this would occur is unclear. Each whisker is attached to the neck by adapter protein gp37, which binds to a gp30 dimer. This is then bound to a dimer of gp31, completing the whisker. One monomer of gp31 in the “down” whiskers interacts directly with the tail sheath protein in the first helical turn of the tail.

A key component of the neck is the dodecameric portal complex. The portal is involved in nucleating capsid assembly and acts as the conduit through which DNA is packaged. At the interface between capsid and tail, it also facilitates the transfer of DNA from the capsid to the tail tube for genome ejection^25–28^. The turbine-shaped portal protein can be subdivided into at least four domain types (Fig. S4, blue text). At the top of the end most internalized into the capsid is the crown domain, followed by the wing, stem, and clip domains^28^. The α-helical crown domain, formed by the C-termini of the twelve portal proteins, is thought to work as a DNA gauge and detect the amount present in the capsid during packaging and start genome translocation^29,30^. The 12 wing domains interact with the internal surface of the capsid, and structural studies suggest this domain is where the first DNA to be packaged resides and becomes anchored to the portal^30^. The stem domain aligns with the capsid shell and has two anti-parallel α-helices that are critical for proper packaging, while the clip domain at the bottom of the portal complex extends to the capsid exterior. The clip domain is where the large terminase DNA packaging motor binds during morphogenesis and where the neck proteins assemble after packaging^30^.

Portal protein structure is remarkably conserved despite divergent sequences with evolutionary ties to gene transfer agents and prophages across diverse genera of bacteria^30^. Despite this conservation, there are a few type structures, such as the HK97-, SPP1-, and lambda-type portal proteins. The Sf14 portal is structurally most similar to that of HK97 and Agrobacterium phage Milano (Fig. S4). Despite several overall similarities between Sf14 and the *T *= 9 virus Anabaena phage A-1(L), their portal proteins are quite different, with A-1(L) having an additional series of β-strands in the wing domain and a barrel atop the crown domain^31^.

### Two sets of tail fibers are retracted on the tail, connected to a simple Mu-like baseplate

Based on initial electron micrographs during characterization^13^, Sf14 and related Moogleviruses appeared to have a set of tail fibers that were often wrapped around the tail, or retracted, with some particles having unwrapped or extended fibers^13,24^. The genome encodes two tail fiber proteins, gp52 and gp53, which are 391 and 792 amino acids in length. Previously, the location and conformation of each were unclear, though there is likely similarity with phage Mu. Mu phages also have two sets of tail fibers, each of which recognizes a different region of lipopolysaccharide (LPS), though only one is expressed at a time depending on the direction of gene expression^32^. These tail fibers, formed by either gp49/gp50 or gp51/gp52, recognize the outer and inner core of LPS, respectively. Previous results showed that Sf14 and its close relatives can infect numerous serotypes of *S. flexneri*^13^, which have variable O-antigen structures at the distal end of LPS^33^ but conserved outer and inner core structures^34^. Shigella have different outer core structures between species but similar inner core structures^34^. We, therefore, hypothesize at least one of the two Sf14 fibers recognizes the outer core, but the binding partner of the second set of fibers is unclear. A related FelixO1-like virus infecting E. coli, vB_EcoM_VpaE1, was also shown to recognize the outer core LPS for infection^35^. This may be a common receptor type across Ounavirinae.

Structurally, some of the best-characterized tail fibers are from T4^36–39^. This myovirus also uses two types of tail fibers: long-tail fibers (LTFs) and short-tail fibers (STFs). As shown in ref. ^39^, the six LTFs are typically wrapped up around the tail in the retracted position, while the six STFs are wrapped around the baseplate. The LTFs periodically extend, with one often being extended at any given time, which facilitates reversible binding with host primary cellular receptors. In the case of T4, this is LPS^39–41^. Once the LTFs bind LPS, the STFs extend and mediate irreversible binding to secondary receptors: for T4, outer membrane protein C (OmpC^39–41^;). Interactions between STF and OmpC bring the central tail fiber or spike closer to the bacterial outer membrane. Rearrangement of the baseplate triggers phage tail contraction, membrane penetration, and subsequent genome ejection into the host cell^39^.

**Fig. 6:**
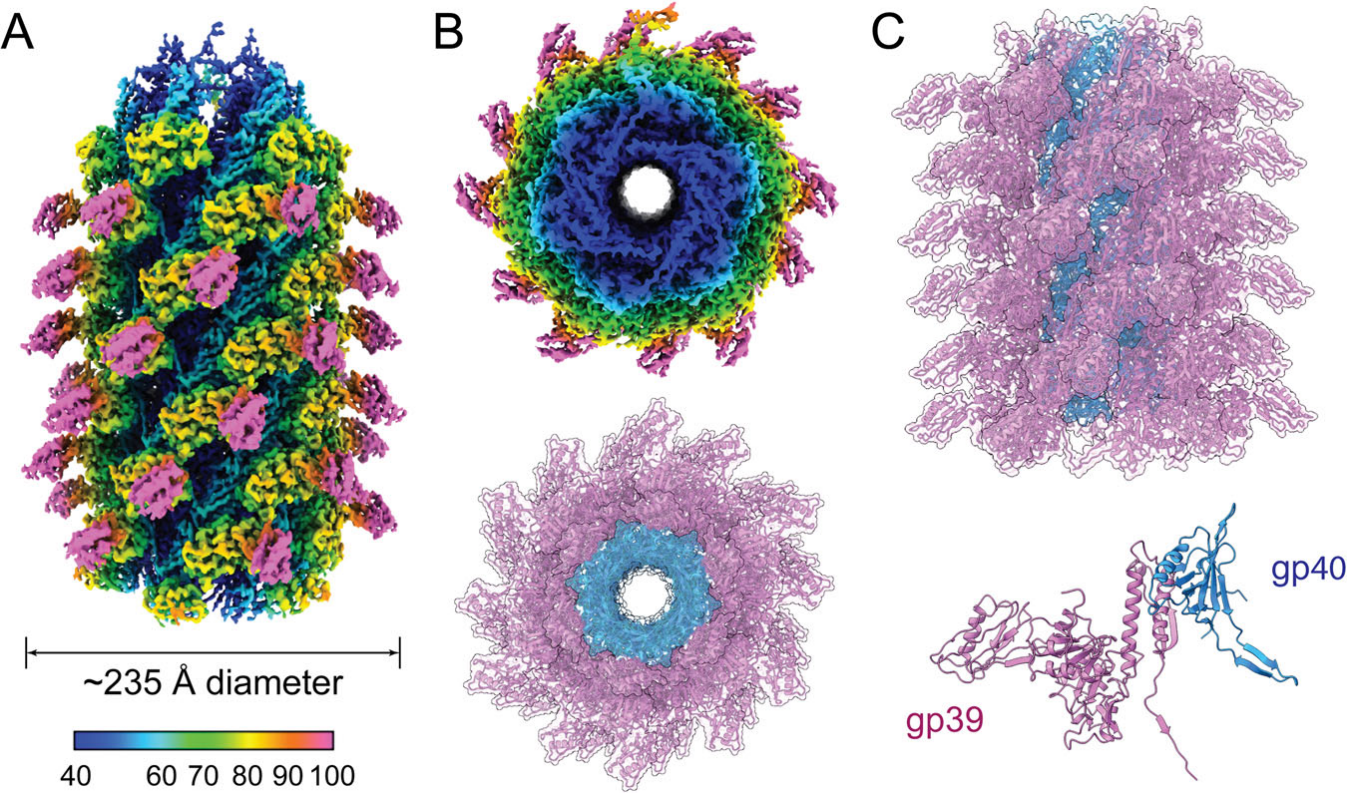
Reconstructions of the Sf14 tail. Helical reconstruction of the tail, with (**A**) full map, (**B**) cross-section of the map (top) and helical model (bottom), and (**C**) side view of the ribbon model, with the asymmetric unit shown below. The colors in (**A**) and (**B**) correspond to radial distance in Å from the center of the tail tube, based on the key at the bottom of the full map.

Here we determined the full structure of the uncontracted, pre-ejection tail, including the tail fiber proteins, baseplate, and central tail needle. A helical reconstruction of the tail is shown in Fig. 6A, with cross-sections showing poorly resolved density in the central tube that could be attributed to tape measure proteins and/or genomic DNA (Fig. 6B). Dimers of the tail sheath and tube proteins (gp39 and gp40) that comprise the helix were also determined by ModelAngelo (Fig. 6C). We next generated a C6 reconstruction of the tail, which indicated Sf14 has two tail fiber proteins (Fig. 6D). Both sets of fibers resemble the long tail fibers of phage Mu and wrap up around the tail in the retracted position.

**Fig. 7:**
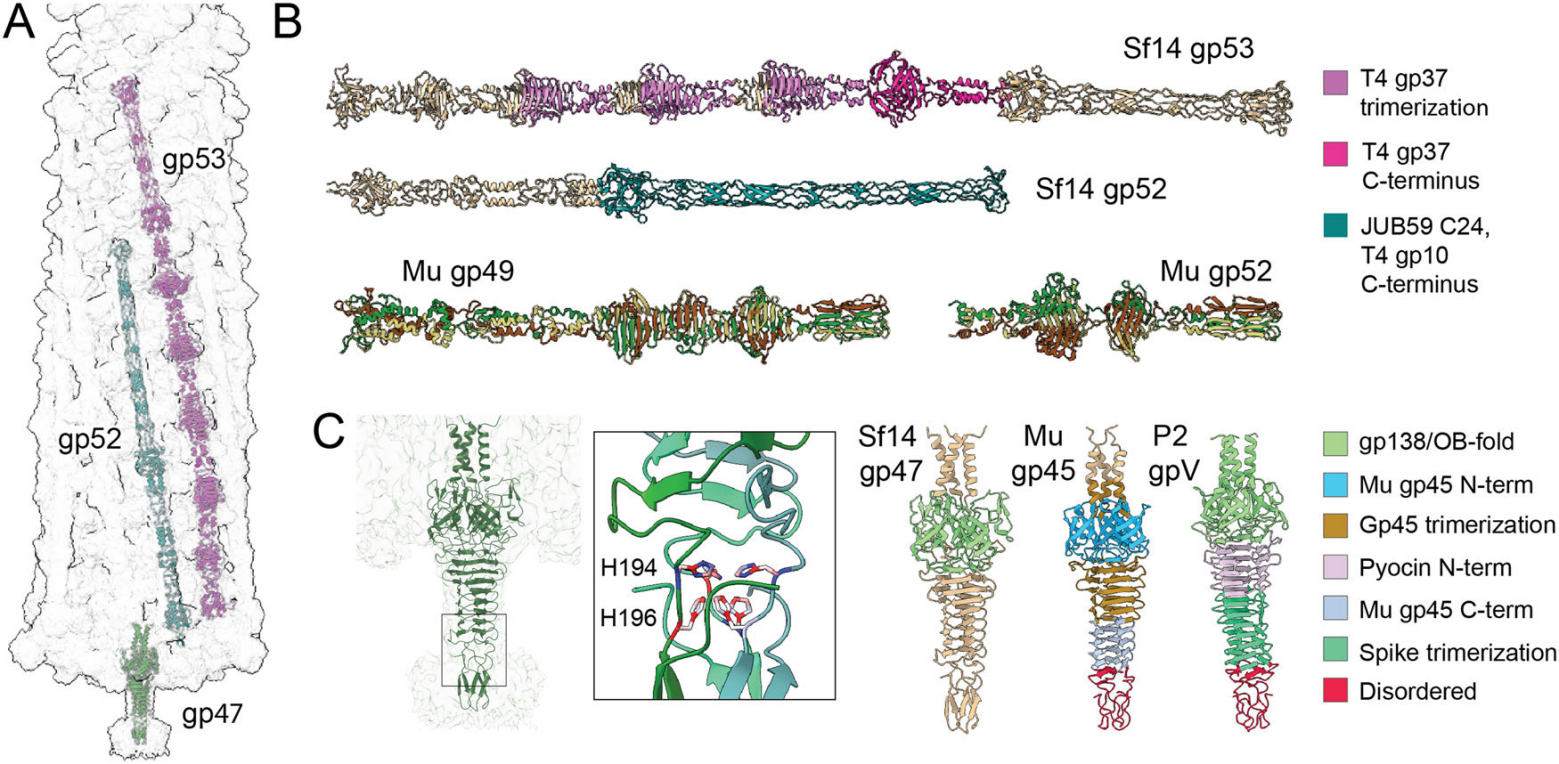
C6 tail reconstruction, including tail fibers and baseplate. **A** AlphaFolded Sf14 proteins gp47 (tail needle) gp52 (short LTF), and gp53 (long LTF) trimers are docked into the tail map using ChimeraX. **B** Comparison between Sf14 tail fiber proteins and their domains as indicated by the colors to the right. The tail fibers of phage Mu colored by chain (gp49, 5YVQ; gp52, 8JU3) are included due to the Mu-like baseplate of Sf14, despite the differences in tail fibers. **C** A focus on the tail needle trimer of Sf14 and its iron binding region (inset), which is predicted to aid in membrane penetration (see Supplementary Text). His 194 from each monomer is labeled H194 to denote the identity of the upper ring of His residues; His 196 from each monomer, H196, corresponds to the lower ring of His residues. On the right, Sf14 gp47 and its homologs were compared based on their domain architecture. The colors each represent a specific domain family as indicated (see also Supplemental Figs. S5–S7 for more detail and additional comparisons).

Using measurements of the cryoEM density and predicted protein structures, we determined that the longest tail fiber is gp53 at 630 Å and the shorter tail fiber is gp52 at approximately 430 Å (Fig. 7A). We also determined the placement of the central needle, gp47. The Sf14 longer tail fiber gp53 contains domains similar to the T7 tailspike gp17 C-terminus and Mu gp49 tail fiber. In T7 gp17, the C-terminus contains a b-sheet pyramid domain and a terminal b-propeller tip domain, the latter of which interacts with *E. coli* lipopolysaccharide^42,43^. Sf14 gp53 does not contain a true b-propeller tip domain, and it is longer than the Mu gp49 fiber by almost 300 amino acids (Fig. 6B top). Within the Ounavirinae, the gp53 structure appears to be conserved across all isolates despite low amino acid identity (Supplemental Fig. S5). For example, the amino acid identity between Sf14 gp53 and its homolog gp56 from type virus Moogle is only 47%. Much of the divergence appears to be at the distal tip, which could signify differences in host range or receptor usage.

The Sf14 shorter tail fiber gp52 contains a C24-like domain and is rich in Ser and Val residues. Protein C24 is the receptor-binding protein of temperate phage *Bizionia argentinensis* JUB59^44^, with some structural similarity to the T4 LTF receptor-binding tip. However, based on both protein sequence and AlphaFold predicted structure, the C24-like domain is again in the center of the fiber rather than at the tip (Fig. 7B bottom). This may reflect a difference between these phages in the orientation of the tail or tail fibers during initial binding, with receptor interactions occurring more proximal than the tip itself. Gp52 is also predicted to contain a T4 gp10 C-terminus-like domain (Supplemental Fig. S5). T4 gp10 is a baseplate protein that connects the short tail fibers to the baseplate. After the initial stages of attachment, gp10 will rotate and act as a lever to extend the short tail fibers, mediating additional contact with the cell surface. In the context of Sf14 gp52, this domain appears to be involved in host attachment rather than baseplate binding or fiber rotation.

The Sf14 baseplate wedge subunit gp49 resembles Mu gp47 and P2 gpJ. In “simple” Mu-like baseplates, this subunit binds two other proteins to form the wedge complex^15^. For Sf14, the wedge complex likely consists of gp48, gp49, and gp50, though the resolution was not high enough to confirm the identities or interactions here. Mu wedges typically have 2:2:2 stoichiometry, with 6:6:6 stoichiometry in the assembled baseplate^15^. Six tail fibers then typically attach to the periphery of the baseplate^15,45^, with one fiber per peripheral subunit or two fibers per wedge. The binding mechanism of greater than six fibers is less clear. For example, myophage Pam3, which has 12 fibers comprised of tail fiber protein gp24 homotrimers, six fibers point up and six-point down, with the up fiber forming disulfide bonds with the peripheral subunit of the baseplate wedge and the down fiber forming disulfide bonds with both an outer and inner subunit of the wedge^46^. Although Sf14 gp52 contains a Cys in the baseplate-binding region (the amino acid sequence for residues 31–36 is Glu-Asp-Arg-Lys-**Cys**-Thr), there are no cysteines in this region of gp53. Instead, there is a Met in place of Cys (the amino acid sequence for residues 35-40 is Glu-Asp-Arg-Lys-**Met**-Thr). Since only the hypothesized central gp49 and peripheral gp50 subunits of the baseplate wedge contain Cys, gp52 likely interacts with gp50 rather than the other peripheral subunit gp48. At this time, it is unclear how gp53 is interacting with the baseplate wedge, but based on stoichiometry it likely binds the non-Cys-containing gp48.

The central hub is a ring made of gp46 connecting to the central spike, a trimer of gp47. The tail needle spans 88.8 Å and has a knob at the tip (Fig. 7C). Like Mu gp45 and P2 gpV tail needles, the Sf14 tail needle appears to have an iron-binding region below the triple β-helix, which contains a conserved double-histidine (HxH) motif (Fig. 7C inset). It is hypothesized that iron serves a stabilizing role during membrane puncturing, ensuring the spike protein remains rigid during membrane translocation. A comparison of the overall structure between these tail needles is also shown in Fig. 7C, right, and Supplemental Fig. S7. Conversely, the apical cap on the Sf14 tail needle does not seem likely to facilitate membrane penetration. This protein may instead be involved in exolysin activity or have some other function during host identification that is currently unclear.

## Discussion

Bacteriophages have been found in numerous environments and display a variety of structures. The tailed phages have icosahedral heads, with most well-characterized phages displaying *T* = 7 or *T* = 13 icosahedral symmetry. Along with phage hunting contributing significantly to our knowledge base of phage diversity, it has also revealed more phages with *T* = 9 symmetry infecting a variety of non-model bacteria. These *T* = 9 capsids are also often decorated with accessory proteins, or decoration proteins, which may serve a variety of functions. Previously described decoration proteins aid in the stability of phages P22 and lambda^47,48^, or they can serve as environmental sensors as in T4^14^. Thus far, decoration proteins also occupy a variety of organizations and orientations^18^. In Sf14, we see two different types of decoration proteins. Gp33 forms a lattice of trimers that is often seen in decoration proteins conferring stability, while gp20 occupies the center of hexamers at the quasi-3 fold axes of symmetry, similar to the environmental-sensing T4 Hoc. These are not typically seen in combination.

Previous studies on *T* = 9 capsids have suggested that the core HK97 fold is sufficient for capsid stabilization^10^. Results shown here are consistent with this hypothesis, as even degraded capsids without genome still contain both decoration proteins. The gp33 decoration proteins may be serving another function, such as host attachment or particle aggregation, or they may stabilize the capsid during DNA packaging but subsequently lose the genome through weakness elsewhere. Additional studies are necessary to investigate these hypotheses. The evolution of Sf14’s two decoration proteins and two tail fibers is unclear but may have arisen due to recombination between phage genes and bacterial prophage genes. There are similarities between these Mooglevirus proteins and related proteins throughout the *Caudoviricetes* class of phages. This could suggest either early origins of these proteins, followed by specialization, or more likely, provides yet another example of rampant horizontal gene transfer between double-stranded DNA phages, and between host and parasite.

In addition to combining two types of decoration proteins, Sf14 also appears to have a hybrid tail structure. The baseplate is structurally similar to many myoviruses, containing a “simple” Mu-like organization. Its tail fibers are also generally similar to the Mu-type tail fibers, but the Sf14 longer tail fiber gp53 appears to have acquired a much longer T4 gp37-like distal tip. In addition, while Mu tail fibers are comprised of two types of proteins—a tail fiber and a chaperone—both Sf14 tail fibers appear to consist of only a single protein. There are also no homologs of the Mu chaperone proteins gp50 and gp51 encoded in the Sf14 genome. The biological implications of having both Mu-like and T4-like tail structures have also not yet been determined.

Overall, phage Sf14 appears to share some similarities to other model phages, but in different combinations: both λ- and T4-like decoration proteins, HK97-like capsid and portal proteins, T4/Mu-like tail fibers, a Mu-like baseplate, and a P2-like tail needle. The diversity of known phages is still being realized, and resolving additional phage structures will help us better determine their form and function, along with their evolutionary relationships. As phages are known to undergo horizontal gene exchange, this will also be important in predicting the biological mechanisms of previously undescribed phages.

## Materials and methods

### Bacteria and phage methods

*S. flexneri* strain CFS100 is an avirulent derivative of 2457 T from the collection of Shelley Payne (UT-Austin) that was cured of its virulence plasmid^49^. Bacteriophage Sf14, along with its growth and plating methods, were conducted as described in ref. ^13^. To summarize, bacteriophage Sf14 was propagated in *S. flexneri* CFS100 by inoculating a liquid LB culture and shaking at 37 °C until lysis. The cell debris was centrifuged at 8000×*g* for 10 min, then the supernatant was collected and subsequently centrifuged at 13,000×*g* for 45 min. The supernatant was discarded, and the pellet was gently resuspended in phage dilution buffer (10 mM Tris, pH 7.6; 10 mM MgCl_2_). Phage stocks were stored at 4 °C. For soft agar overlay, phage and 150 μL of host bacteria were mixed with 2 mL soft agar (0.65% agar, 0.65% nutrient broth, 0.5% NaCl), then poured over an agar plate.

### Mass spectrometry

To determine proteins present in the Sf14 particle, an aliquot of approximately 1 × 10^11^ PFU was TCA precipitated and then run on a 12% SDS-PAGE gel under denaturing conditions. When the sample had just entered the separation portion of the gel, the band was then cut and sent to the University of Florida Proteomics and Mass Spectrometry Core. The sample was washed once with buffer, treated with 10 mM TCEP and 2-chloroacetamide, and digested with trypsin/lys-C overnight. Peptides were eluted, lyophilized, and desalted via ZipTip. The sample was then analyzed on a timsTOF fleX MS and compared to the proteome of Sf14 and *S. flexneri* 2457 T, which is a close relative of the host strain CFS100.

### Cryo-electron microscopy sample preparation, data collection, and processing

Small (3–5 μL) aliquots of phage concentrated to ~10^12^ PFU/mL were applied to R2/2 Quantifoil grids (Electron Microscopy Solutions) that had been glow discharged for 45 s in a Pelco Easiglow glow discharging unit. The samples were plunge frozen in liquid ethane using a Vitrobot Mark IV operated at 4 °C and 100% humidity, with a blot force of 1 and 5 s of blotting time per grid. Data were collected at the Purdue Cryo-EM facility using a Titan Krios equipped with a K3 direct electron detector operating at 300 keV with a post-column GIF (20 eV slit width) under low dose conditions. Movies were collected at 53,000× nominal magnification (0.816 Å/pixel) by recording 40 frames over 4.4 s for a total dose of 33 e^−^/Å^2^.

A total of 6788 micrographs were recorded and all subsequent processing was carried out in CryoSPARC v4.4.1^50^. The micrographs were first motion corrected using Patch Motion correction, followed by CTF estimation using Patch CTF estimation. After curation based on CTF fit resolution, 6637 micrographs were used for further processing in Relion v3.1.1^51^. The resulting volume was used in CryoSPARC for both generating templates for particle picking and as an initial model for refinement. After template picking and 2D classification 42,269 full capsid particles and 98,511 empty capsid particles were selected for refinement. 3D refinement with I1 symmetry was used for both the full and empty capsid particles with the previously obtained map as an initial volume.

An asymmetric reconstruction to resolve the unique tail vertex was done via symmetry relaxation with marginalization of full capsids. The center of the neck was then boxed and a total of 37,006 particles were extracted. The neck was reconstructed with alignment parameters from symmetry relaxation, then refinement was completed with C6 symmetry resulting in a 3.4 Å resolution map. The center of the box from the neck reconstruction was shifted to cover the initial part of the tail tube connecting the neck and base plate, as the neck reconstruction resolved parts of the central tail tube. Refinement was carried out without applying any helical parameters or point group symmetry. The resulting map was then used to determine helical symmetry parameters using the CryoSPARC symmetry search utility. Helical refinement was then conducted with C6 point group symmetry and helical parameters (Rise = 37.062 Å, Twist = 25.750°) resulting in a map of 3.4 Å resolution.

For the C6 reconstructions of the whole tail, first, the center of the box on the helical reconstruction was shifted further down the tail tube particles were extracted, and a helical reconstruction was enacted. The resulting volume did not include the base plate due to box size restraints, but templates generated from this volume were used to facilitate a round of template picking to select whole tail particles. After 2D classification, a total of 11,590 particles were used for refinement with C6 symmetry resulting in a map of 4.1 Å resolution. The center of the box was then shifted to the center of the base plate and a total of 11,474 particles were extracted. The extracted particles were reconstructed with alignment parameters from the whole tail reconstruction, and refinement was achieved with C6 symmetry, resulting in a map of 4.0 Å resolution. All resolutions were estimated based on the Fourier shell correlation cut-off of 0.143. Data collection and processing statistics for all maps are listed in Table 2.

**Table 2:** Data collection and processing statistics for all six maps, including their respective EMDB and PDB accession numbers

Data Collection and Processing	Virion	Empty Virion	Neck	Tail (helical)	Whole Tail	Baseplate
EMDB ID	EMD-45155	EMD-45163	EMD-45162	EMD-45164	EMD-45168	EMD-45169
PDB ID	9C2D	9C3A	9C39	9C3B	–	–
# micrographs	6788	6788	6788	6788	6788	6788
# final particles	42,269	98,511	37,006	23,422	11,590	11,474
Symmetry imposed	I1	I1	C6	C6, Rise 37.062 Å Twist 25.750°	C6	C6
Å Resolution (FSC_0.143_)	3.2	3.1	3.4	3.4	4.1	4
Contour	0.584	0.824	0.244	0.683	0.683	0.178

### Model building and refinement

Individual structures of the decoration protein gp33, capsid protein gp34, and tail proteins gp52, gp53, and gp47 were predicted via AlphaFold^52,53^. Other initial models were generated with ModelAngelo^17^. Models were docked into EM maps using UCSF Chimera (version 1.15) and UCSF ChimeraX (version 1.8)^54,55^, then adjusted using Coot (version 0.9.7)^56,57^. These were subsequently refined using several iterative rounds of Phenix (version 1.20.2)^58^ RealSpace Refine^59^, with the final model evaluated by MolProbity^60^. Refinement statistics are shown in Table 3.

**Table 3:** Model refinement statistics for the virion, empty virion, neck, and tail asymmetric units

Refinement Stats	Virion	Empty Virion	Neck	Tail (helical)
PDB ID	9C2D	9C3A	9C39	9C3B
*Bonds (RMSD)*				
Length (Å)	0.004 (3)	0.009 (244)	0.004 (0)	0.007 (0)
Angles (°)	0.736 (51)	1.467 (578)	0.835 (11)	0.869 (7)
MolProbity score	2.08	2.17	2.16	2.41
Clash score	9.28	9.05	11.35	10.52
Rotamer outliers (%)	1.95	2.74	1.18	2.78
*Ramachandran plot (%)*				
Outliers	0.67	0.76	0.79	1.38
Allowed	4.72	4.29	8.59	8.09
Favored	94.61	94.95	90.62	90.53
CC (volume)	0.87	0.84	0.86	0.89

### Comparative structural analyses

Once all structural proteins were determined, they were analyzed by BLAST, psiBLAST, and DALI^19^ to identify closest relatives based on protein sequence or structure. Conserved domains and signatures of Sf14 proteins and their relatives were analyzed by InterProScan^61^. Representative-related protein structures were predicted by AlphaFold^62^ if empirically determined structures were not available. All proteins were visualized and compared in UCSF ChimeraX^55^. Sequences of model phage proteins or closely related proteins were used to build phylogenetic trees, which were generated on Phylogeny.fr via MUSCLE alignment, Gblocks curation, and MrBayes construction, then visualized using TreeDyn^63^.

### Statistics and reproducibility

Statistics for data collection and processing, including sample sizes, are provided in Tables 2 and 3.

### Reporting summary

Further information on research design is available in the Nature Portfolio Reporting Summary linked to this article.

## Supplementary information

Supplemental Material

Reporting summary

## Data Availability

EMDB and PDB accession numbers are as follows: virion (EMD-45155, PDB 9C2D), empty virion (EMD-45163, PDB 9C3A); neck (EMD-45162, PDB 9C39); helical tail (EMD-45164, PDB 9C3B), C6 tail (EMD-45168), and baseplate (EMD-45169). Mass spectrometry spectra and results are available in MassIVE (accession number MSV000096931, 10.25345/C5Z892S9V). Statistics and accession numbers can be found in Tables 2 and 3. Additional information, including a representative micrograph and further comparative studies, can be found in the Supplementary File. Materials and data are available upon request.

## References

[1] Cobian Guemes, A. G. et al. Viruses as winners in the game of life. *Annu. Rev. Virol.* **3**, 197–214 (2016). 27741409 10.1146/annurev-virology-100114-054952

[2] Jordan, T. C. et al. A broadly implementable research course in phage discovery and genomics for first-year undergraduate students. *mBio* **5**, e01051–01013 (2014). 24496795 10.1128/mBio.01051-13 PMC3950523

[3] Dion, M. B., Oechslin, F. & Moineau, S. Phage diversity, genomics and phylogeny. *Nat. Rev. Microbiol.* **18**, 125–138 (2020). 32015529 10.1038/s41579-019-0311-5

[4] Hua, J. et al. Capsids and genomes of jumbo-sized bacteriophages reveal the evolutionary reach of the HK97 fold. *mBio*10.1128/mBio.01579-17 (2017). 10.1128/mBio.01579-17 PMC5646251 29042498

[5] Choi, K. H. et al. Insight into DNA and protein transport in double-stranded DNA viruses: the structure of bacteriophage N4. *J. Mol. Biol.* **378**, 726–736 (2008). 18374942 10.1016/j.jmb.2008.02.059 PMC2396777

[6] Grose, J. H. et al. The genomes, proteomes, and structures of three novel phages that infect the Bacillus cereus group and carry putative virulence factors. *J. Virol.* **88**, 11846–11860 (2014). 25100842 10.1128/JVI.01364-14 PMC4178739

[7] Cui, N. et al. Capsid structure of anabaena cyanophage A-1(L). *J. Virol.* **95**, e0135621 (2021). 34549983 10.1128/JVI.01356-21 PMC8610606

[8] Warring, S. L. et al. A lipopolysaccharide-dependent phage infects a pseudomonad phytopathogen and can evolve to evade phage resistance. *Environ. Microbiol.* **24**, 4834–4852 (2022). 35912527 10.1111/1462-2920.16106 PMC9796965

[9] Zheng, J. et al. A Capsid Structure of *Ralstonia solanacearum* podoviridae GP4 with a triangulation number *T* = 9. *Viruses*10.3390/v14112431 (2022). 10.3390/v14112431 PMC9698820 36366529

[10] Podgorski, J. M. et al. A structural dendrogram of the actinobacteriophage major capsid proteins provides important structural insights into the evolution of capsid stability. *Structure* **31**, 282–294.e285 (2023). 36649709 10.1016/j.str.2022.12.012 PMC10071307

[11] Martin-Bravo, M., Llorente, J. M. G., Hernandez-Rojas, J. & Wales, D. J. Minimal design principles for icosahedral virus capsids. *ACS Nano* **15**, 14873–14884 (2021). 34492194 10.1021/acsnano.1c04952 PMC8939845

[12] Podgorski, J. et al. Structures of three actinobacteriophage capsids: roles of symmetry and accessory proteins. *Viruses*10.3390/v12030294 (2020). 10.3390/v12030294 PMC7150772 32182721

[13] Doore, S. M., Schrad, J. R., Dean, W. F., Dover, J. A. & Parent, K. N. Shigella Phages Isolated during a Dysentery Outbreak Reveal Uncommon Structures and Broad Species Diversity. *J. Virol.*10.1128/JVI.02117-17 (2018). 10.1128/JVI.02117-17 PMC5874400 29437962

[14] Fokine, A. et al. Structure and function of Hoc-A novel environment sensing device encoded by T4 and other bacteriophages. *Viruses*10.3390/v15071517 (2023). 10.3390/v15071517 PMC10385173 37515203

[15] Buttner, C. R., Wu, Y., Maxwell, K. L. & Davidson, A. R. Baseplate assembly of phage Mu: Defining the conserved core components of contractile-tailed phages and related bacterial systems. *Proc. Natl Acad. Sci. USA* **113**, 10174–10179 (2016). 27555589 10.1073/pnas.1607966113 PMC5018775

[16] Mori, Y. et al. Determination of the three-dimensional structure of bacteriophage Mu(-) tail fiber and its characterization. *Virology* **593**, 110017 (2024). 38382161 10.1016/j.virol.2024.110017

[17] Jamali, K. et al. Automated model building and protein identification in cryo-EM maps. *Nature* **628**, 450–457 (2024). 38408488 10.1038/s41586-024-07215-4 PMC11006616

[18] Dedeo, C. L., Teschke, C. M. & Alexandrescu, A. T. Keeping It Together: Structures, Functions, and Applications of Viral Decoration Proteins. *Viruses*10.3390/v12101163 (2020). 10.3390/v12101163 PMC7602432 33066635

[19] Holm, L. Dali server: structural unification of protein families. *Nucleic Acids Res.* **50**, W210–W215 (2022). 35610055 10.1093/nar/gkac387 PMC9252788

[20] Potapov, V., Sobolev, V., Edelman, M., Kister, A. & Gelfand, I. Protein–protein recognition: juxtaposition of domain and interface cores in immunoglobulins and other sandwich-like proteins. *J. Mol. Biol.* **342**, 665–679 (2004). 15327963 10.1016/j.jmb.2004.06.072

[21] Taheri, M. et al. Self recognition in the Ig superfamily. Identification of precise subdomains in carcinoembryonic antigen required for intercellular adhesion. *J. Biol. Chem.* **275**, 26935–26943 (2000). 10864933 10.1074/jbc.M909242199

[22] Tang, H., Finn, R. D. & Thomas, P. D. TreeGrafter: phylogenetic tree-based annotation of proteins with Gene Ontology terms and other annotations. *Bioinformatics* **35**, 518–520 (2019). 30032202 10.1093/bioinformatics/bty625 PMC6361231

[23] Effantin, G., Boulanger, P., Neumann, E., Letellier, L. & Conway, J. F. Bacteriophage T5 structure reveals similarities with HK97 and T4 suggesting evolutionary relationships. *J. Mol. Biol.* **361**, 993–1002 (2006). 16876823 10.1016/j.jmb.2006.06.081

[24] Doore, S. M. et al. A cornucopia of Shigella phages from the Cornhusker state. *Virology* **538**, 45–52 (2019). 31569014 10.1016/j.virol.2019.09.007 PMC7009021

[25] Fokine, A. & Rossmann, M. G. Molecular architecture of tailed double-stranded DNA phages. *Bacteriophage* **4**, e28281 (2014). 24616838 10.4161/bact.28281 PMC3940491

[26] Parent, K. N., Schrad, J. R. & Cingolani, G. Breaking symmetry in viral icosahedral capsids as seen through the lenses of X-ray crystallography and cryo-electron microscopy. *Viruses*10.3390/v10020067 (2018). 10.3390/v10020067 PMC5850374 29414851

[27] Prevelige, P. E. Jr. & Cortines, J. R. Phage assembly and the special role of the portal protein. *Curr. Opin. Virol.* **31**, 66–73 (2018). 10.1016/j.coviro.2018.09.004 30274853

[28] Dedeo, C. L., Cingolani, G. & Teschke, C. M. Portal protein: the orchestrator of capsid assembly for the dsDNA tailed bacteriophages and herpesviruses. *Annu. Rev. Virol.* **6**, 141–160 (2019). 31337287 10.1146/annurev-virology-092818-015819 PMC6947915

[29] Olia, A. S., Prevelige, P. E. Jr., Johnson, J. E. & Cingolani, G. Three-dimensional structure of a viral genome-delivery portal vertex. *Nat. Struct. Mol. Biol.* **18**, 597–603 (2011). 21499245 10.1038/nsmb.2023 PMC3087855

[30] Rao, V. B., Fokine, A. & Fang, Q. The remarkable viral portal vertex: structure and a plausible model for mechanism. *Curr. Opin. Virol.* **51**, 65–73 (2021). 34619513 10.1016/j.coviro.2021.09.004 PMC9579958

[31] Yu, R. C. et al. Structure of the intact tail machine of Anabaena myophage A-1(L). *Nat. Commun.* **15**, 2654 (2024). 38531972 10.1038/s41467-024-47006-z PMC10966104

[32] Van de Putte, P., Cramer, S. & Giphart-Gassler, M. Invertible DNA determines host specificity of bacteriophage mu. *Nature* **286**, 218–222 (1980). 6250048 10.1038/286218a0

[33] Knirel, Y. A, et al. O-antigen modifications providing antigenic diversity of Shigella flexneri and underlying genetic mechanisms. Biochemistry 80, 901–914–914 (2015). 26542003 10.1134/S0006297915070093

[34] Knirel, Y. A. et al. Lipopolysaccharide core structures and their correlation with genetic groupings of Shigella strains. A novel core variant in *Shigella boydii* type 16. *Glycobiology* **21**, 1362–1372 (2011). 21752864 10.1093/glycob/cwr088

[35] Simoliunas, E. et al. Incomplete LPS core-specific felix01-like virus vB_EcoM_VpaE1. *Viruses* **7**, 6163–6181 (2015). 26633460 10.3390/v7122932 PMC4690856

[36] Hyman, P. & van Raaij, M. Bacteriophage T4 long tail fiber domains. *Biophys. Rev.* **10**, 463–471 (2018). 29204885 10.1007/s12551-017-0348-5 PMC5899708

[37] Islam, M. Z. et al. Molecular anatomy of the receptor binding module of a bacteriophage long tail fiber. *PLoS Pathog.* **15**, e1008193 (2019). 31856258 10.1371/journal.ppat.1008193 PMC6957217

[38] Leiman, P. G. et al. Morphogenesis of the T4 tail and tail fibers. *Virol. J.* **7**, 355 (2010). 21129200 10.1186/1743-422X-7-355 PMC3004832

[39] Hu, B., Margolin, W., Molineux, I. J. & Liu, J. Structural remodeling of bacteriophage T4 and host membranes during infection initiation. *Proc. Natl Acad. Sci. USA* **112**, E4919–E4928 (2015). 26283379 10.1073/pnas.1501064112 PMC4568249

[40] Washizaki, A., Yonesaki, T. & Otsuka, Y. Characterization of the interactions between *Escherichia coli* receptors, LPS and OmpC, and bacteriophage T4 long tail fibers. *Microbiologyopen* **5**, 1003–1015 (2016). 27273222 10.1002/mbo3.384 PMC5221442

[41] Yu, F. & Mizushima, S. Roles of lipopolysaccharide and outer membrane protein OmpC of Escherichia coli K-12 in the receptor function for bacteriophage T4. *J. Bacteriol.* **151**, 718–722 (1982). 7047495 10.1128/jb.151.2.718-722.1982 PMC220313

[42] Garcia-Doval, C. & van Raaij, M. J. Structure of the receptor-binding carboxy-terminal domain of bacteriophage T7 tail fibers. *Proc. Natl Acad. Sci. USA* **109**, 9390–9395 (2012). 22645347 10.1073/pnas.1119719109 PMC3386108

[43] Gonzalez-Garcia, V. A. et al. Conformational changes leading to T7 DNA delivery upon interaction with the bacterial receptor. *J. Biol. Chem.* **290**, 10038–10044 (2015). 25697363 10.1074/jbc.M114.614222 PMC4400320

[44] Pellizza, L. et al. Structure of the putative long tail fiber receptor-binding tip of a novel temperate bacteriophage from the Antarctic bacterium *Bizionia argentinensis* JUB59. *J. Struct. Biol.* **212**, 107595 (2020). 32736071 10.1016/j.jsb.2020.107595

[45] Taylor, N. M. I., van Raaij, M. J. & Leiman, P. G. Contractile injection systems of bacteriophages and related systems. *Mol. Microbiol.* **108**, 6–15 (2018). 29405518 10.1111/mmi.13921

[46] Yang, F. et al. Fine structure and assembly pattern of a minimal myophage Pam3. *Proc. Natl Acad. Sci. USA* **120**, e2213727120 (2023). 36656854 10.1073/pnas.2213727120 PMC9942802

[47] Gilcrease, E. B., Winn-Stapley, D. A., Hewitt, F. C., Joss, L. & Casjens, S. R. Nucleotide sequence of the head assembly gene cluster of bacteriophage L and decoration protein characterization. *J. Bacteriol.* **187**, 2050–2057 (2005). 15743953 10.1128/JB.187.6.2050-2057.2005 PMC1064062

[48] Hernando-Perez, M., Lambert, S., Nakatani-Webster, E., Catalano, C. E. & de Pablo, P. J. Cementing proteins provide extra mechanical stabilization to viral cages. *Nat. Commun.* **5**, 4520 (2014). 25072871 10.1038/ncomms5520

[49] Marman, H. E., Mey, A. R. & Payne, S. M. Elongation factor P and modifying enzyme PoxA are necessary for virulence of *Shigella flexneri*. *Infect. Immun.* **82**, 3612–3621 (2014). 24935977 10.1128/IAI.01532-13 PMC4187845

[50] Punjani, A., Rubinstein, J. L., Fleet, D. J. & Brubaker, M. A. cryoSPARC: algorithms for rapid unsupervised cryo-EM structure determination. *Nat. Methods* **14**, 290–296 (2017). 28165473 10.1038/nmeth.4169

[51] Zivanov, J. et al. New tools for automated high-resolution cryo-EM structure determination in RELION-3. *Elife*10.7554/eLife.42166 (2018). 10.7554/eLife.42166 PMC6250425 30412051

[52] Jumper, J. et al. Highly accurate protein structure prediction with AlphaFold. *Nature* **596**, 583–589 (2021). 34265844 10.1038/s41586-021-03819-2 PMC8371605

[53] Mirdita, M. et al. ColabFold: making protein folding accessible to all. *Nat. Methods* **19**, 679–682 (2022). 35637307 10.1038/s41592-022-01488-1 PMC9184281

[54] Pettersen, E. F. et al. UCSF Chimera—a visualization system for exploratory research and analysis. *J. Comput Chem.* **25**, 1605–1612 (2004). 15264254 10.1002/jcc.20084

[55] Pettersen, E. F. et al. UCSF ChimeraX: Structure visualization for researchers, educators, and developers. *Protein Sci.* **30**, 70–82 (2021). 32881101 10.1002/pro.3943 PMC7737788

[56] Emsley, P. & Cowtan, K. Coot: model-building tools for molecular graphics. *Acta Crystallogr. D Biol. Crystallogr.* **60**, 2126–2132 (2004). 15572765 10.1107/S0907444904019158

[57] Casanal, A., Lohkamp, B. & Emsley, P. Current developments in Coot for macromolecular model building of electron cryo-microscopy and crystallographic data. *Protein Sci.* **29**, 1069–1078 (2020). 31730249 10.1002/pro.3791 PMC7096722

[58] Liebschner, D. et al. Macromolecular structure determination using X-rays, neutrons and electrons: recent developments in Phenix. *Acta Crystallogr. D Struct. Biol.* **75**, 861–877 (2019). 31588918 10.1107/S2059798319011471 PMC6778852

[59] Afonine, P. V. et al. Real-space refinement in PHENIX for cryo-EM and crystallography. *Acta Crystallogr. D Struct. Biol.* **74**, 531–544 (2018). 29872004 10.1107/S2059798318006551 PMC6096492

[60] Chen, V. B. et al. MolProbity: all-atom structure validation for macromolecular crystallography. *Acta Crystallogr. D Biol. Crystallogr.* **66**, 12–21 (2010). 20057044 10.1107/S0907444909042073 PMC2803126

[61] Paysan-Lafosse, T. et al. InterPro in 2022. *Nucleic Acids Res.* **51**, D418–D427 (2023). 36350672 10.1093/nar/gkac993 PMC9825450

[62] Abramson, J. et al. Accurate structure prediction of biomolecular interactions with AlphaFold 3. *Nature* **630**, 493–500 (2024). 38718835 10.1038/s41586-024-07487-w PMC11168924

[63] Dereeper, A. et al. Phylogeny.fr: robust phylogenetic analysis for the non-specialist. *Nucleic Acids Res.* **36**, W465–W469 (2008). 18424797 10.1093/nar/gkn180 PMC2447785

