# Supplementary material for: The structure of Shigella virus Sf14 reveals the presence of two decoration proteins and two long tail fibers
Source: Commun Biol. 2025 Feb 12;8:222. doi: 10.1038/s42003-025-07668-x (PMC11821841; doi:10.1038/s42003-025-07668-x)
Supplement: Supplementary file 1 — Supplemental Material [file 42003_2025_7668_MOESM1_ESM.pdf]

Supplemental Text

Additional comparative analysis of capsid protein gp34

To compare Sf14 gp34 with a capsid protein more similar than HK97, we queried representative structures in the PDB database using the DALI server. Results indicated the most similar structure was the YSD1 bacteriophage capsid protein gp17 with an overall RMSD of 3.3 Å (PDB 6XGQ [1]). Here, the A-domain also significantly differs between the two phages, with YSD1 having an extended A-loop similar to HK97. In multimeric form, this leads to differences at the center of capsomeres, as shown in the hexamer in Supplemental Figure S2. The YSD1 gp17 A-loops overlap extensively at the center, which is lacking in Sf14 gp34.

Decoration protein gp33 trimers and gp20 are present in the empty capsid

There are numerous hypothesized functions for the proteins decorating phage capsids, one of which is to provide additional stability. Since one potential reason T=9 capsids were previously rare could be due to low stability and therefore low recovery, it is possible that Sf14 decoration proteins are involved in maintaining the stability of its T=9 capsid. With numerous empty particles in a typical phage preparation (as shown in Supplemental Figure S1A), we hypothesized these empty particles were partially degraded capsids that lacked at least one type of decoration protein.

To examine the composition of empty capsids in greater detail, we generated a separate reconstruction using only empty particles. This map, also resolved to 3.3 Å, was used to build a model of the capsid, which is shown in Supplemental Figure 3A. The asymmetric subunits were then compared to determine differences in organization or conformation. In contrast to our expectations, the subunit was nearly identical to the virion subunit (Supplemental Figure 3B), with an overall root mean square deviation (RMSD) of 0.437 Å. While there are minor differences in the side chains between interacting trimers and at the interface with gp34, these did not produce measurable shifts to suggest the capsids had lost the decoration proteins or undergone significant conformational changes. Similarly, the interactions between the gp20 capsid-binding domain and the capsid proteins were unchanged between full and empty capsid models.

Additional comparative analyses of tail fiber proteins gp52 and gp53, plus tail needle gp47

Bacteriophage tail fiber proteins recognize and bind to specific host receptors, serving as key determinants of the phage's host range. Tail fibers can have proximal and distal halves where the distal tip interacts with host receptors. All the trimeric tail fiber proteins in Supplemental Figure S4 contain the T4 gp10 C terminus-like domains. T4 gp10 acts as a lever, rotating to extend the short tail fibers and aid in attachment [2,3]. The baseplate structural protein Gp9/Gp10 family represents T4 gp10 N-terminus domain and T4 baseplate protein gp9. Gp9 is a connector protein between the long tail fibers and baseplate and initiates tail contraction after the virus attaches to the host cell [4]. Gp10 is trimeric, with each monomer contains three domains. The N-terminal domain has two alpha helices and an extended polypeptide chain, followed by a long and flexible region, then a seven stranded β sandwich, and ending with the C-terminus domain that resembles a jelly roll fold [2]. Gp10 works with gp11 to initiate baseplate wedge assembly [3]. Interestingly, the phage Ld25A encodes receptor-binding protein gp20, which contains a prophage tail endopeptidase domain, a gp10 C-terminal domain, and tail spike domains. This last domain type is found in the *Bacillus* phage SPP1 tail spike protein gp21, which is a singular central spike at the tip of the tail. Upon binding the *Bacillus* receptor YueB, gp21 undergoes a conformational change cascade that starts at the distal C-terminus and disassembles the tail spike, ultimately opening the cap bound to the gp21 N-terminus [5].

When comparing tail fibers related to Sf14 gp53, each contains at least one T4 long tail fiber gp37 trimer domain (Supplemental Figure S5, colored pink). The gp37 region of the long tail fiber is also known as the distal region, which is involved in binding to receptors on the bacterial surface [6]. Some representative structures shown in Supplemental Figure S5 also contain the gp37 C-terminal domain (shown in dark pink), which is found in bacteria, prophages, and tailed phages and often is grouped alongside the bacteriophage T7 gp17 C-terminal domain in Pfam (e.g. Q38519_BPT7). The T7 tail complex has six kinked tail fibers made of gp17 oligomers, which contain an N-terminal tail-binding domain, a proximal half-fiber domain, and a distal half-fiber domain at the C-terminus [7,8]. This last domain can be divided into pyramid and tip domains, with the pyramid resembling the T4 gp37 C-terminal domain and the tip forming a knob that interacts with the host receptor.

Interestingly, the distal region of gp53 does not share the same domain type as its homologs based on an InterProScan analysis. For example, phage vB_EcoM_AYO145A gp78 has an Ur-lambda gp27-like tail fiber structural domain which has been implicated in binding to an additional outer membrane receptor, thereby increasing the adsorption rate into the host cell [9,10]. This gp27-like tail fiber domain is instead on the other Sf14 long tail fiber, gp52, along with the T4 gp10 C-terminal-like domain. It is possible that both tail fibers are involved in binding two types of host receptors, with gp53 interacting with lipopolysaccharide and gp52 interacting with a protein receptor. Alternatively, as in phage Mu, they could each be responsible for binding different types of lipopolysaccharide on different host cells [11].

A comparison between the Sf14 central tail needle gp47 and other myovirus tail needles is shown in Supplemental Figure S6. Starting at the top or baseplate side of the protein, the domains Gp138_N (IPR041599) and Vgr_OB-fold_dom_sf (IPR037026) are seen in most tail needle structures. Gp138_N refers to the N-terminal domain of Escherichia phage phi92's gp138, which is a spike trimer of interconnected chains. Each chain has an oligosaccharide/oligonucleotide-binding (OB)-fold, with Greek key topology, a 5-stranded antiparallel-barrel with a disulfide bond, and beta-helical C-terminal domain [12]. Phage P2 gpV-related sequences are seen in many bacteriophages and bacteriocins, which are phage tail-derived proteins that kill other host strain-related bacteria by puncturing the membrane. P2 gene V and phi92 gp138-like domains are typically involved in penetration or lysis of the host bacterial cell membrane [12]. This superfamily also includes the Vgr-related proteins like type VI secretion systems (T6SS) found in Gram-negative pathogenic bacteria. VgrG-like proteins are predicted to assemble into a trimeric complex similar to the trimeric tail spike proteins gp27 and gp5 from *E. coli* phage T4 that create a puncturing device to move effector protein domains past target host cell membranes [13]. VgrGs have a conserved region equivalent to the Gp5 OB-fold domain from phage T4 baseplate tail spike complex (shown in Supplemental Figure S6). The domains of Gp5 are connected through long linkers of an N-terminal Ob-fold domain, a lysozyme domain in the middle, and a triple stranded-helix at the C-terminus [14]. The specific entry for this family covers the OB fold part of the structure and it contains an extra beta hairpin that acts as the core for the beta helix in the spike protein [12,15]. The P2 gene V and phi92 gp138-like domain superfamily was found in most phage proteins represented in Figure S6.

The central region of most tail needles contain either the spike trimer domain or the gp138 C-terminal-like domain. Though slightly different, both domains contain long trimeric beta helices are made of three antiparallel beta-sheets that swap their C-terminus beta strands with each other across the threefold axis of symmetry (Supplemental Figure S6). At the very tip, there tends to be a disordered region, for example the gpV-like apex motif, which is found at the C-terminus of the gpV spike protein. Either within this apex region or at the junction between the spike trimer domain and the apex domain is a conserved iron ion-coordinating histidine-x-histidine (HXH) motif; for Sf14, this motif is also shown in main text Figure 7. These "iron-loaded spikes" facilitate the piercing the host membrane [12,15]. Perhaps due to the role of iron in membrane penetration, the phage BCSR129 needle is structurally similar to the other domains discussed above, but InterProScan identifies it as an iron-binding structure.

Other well-characterized tail needle structures include Mu gp45 and T4 gp5. The N-terminal Mu gp45 spike domain has a OB-like β-barrel fold that acts like a distal plug of the tail tube channel, with the C-terminal end used to penetrate the host cell membrane. Mu gp45 is loaded with both iron and calcium ions, but unlike P2 and phi92, only calcium appears to affect binding and infection [16,17]. The T4 gp5 tail needle binds tail protein gp27 via an OB-fold domain at the N-terminus, followed by a lysozyme domain and ending with a β-helix domain. The lysozyme domain is involved in breaking down the peptidoglycan in cell walls, facilitating membrane penetration and genome ejection into the host. It hydrolyses 1,4-β linkages between the N-acetyl-D-glucosamine and N-acetylmuramic acids in cell wall peptidoglycan heteropolymers [18,19]. The T4 gp5 C-terminus binds gp5.4 (not shown), a needle-like β solenoid portion.

Overall, the Sf14 gp47 central tail needle appears to be similar to many other tail needles and to the P2 gpV, with some variation. The trimerization domain is shorter, and the distal tip contains a total of six β-strands rather than the disordered region found in other tail needles. Any biological significance of this structure, in addition to the identity of the apical cap, are currently unclear.

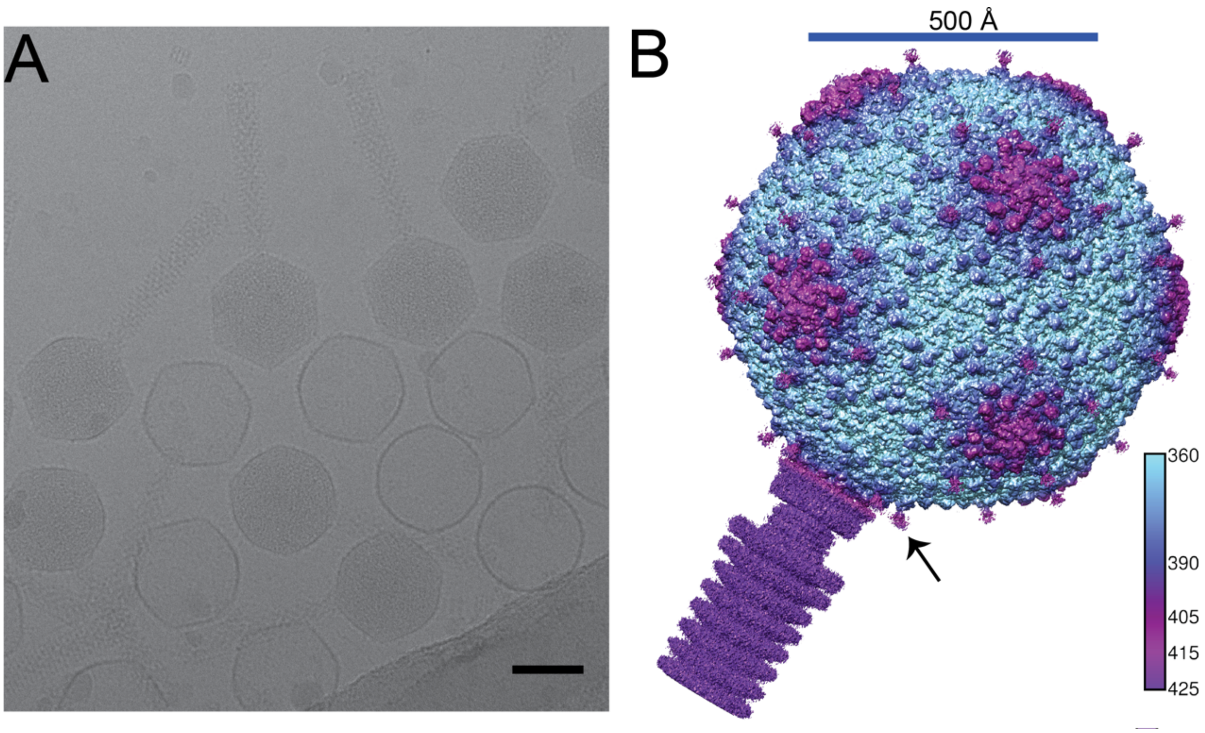

Supplemental Figure S1. A) a representative micrograph showing both full (dark) and empty (light) particles; B) the asymmetric reconstruction of the Sf14 capsid at 4.5 Å on its own, rather than as part of a composite image shown in main text Figure 1. The protruding decoration protein gp20 is indicated with an arrow. The scale bar in A represents 50 nm.

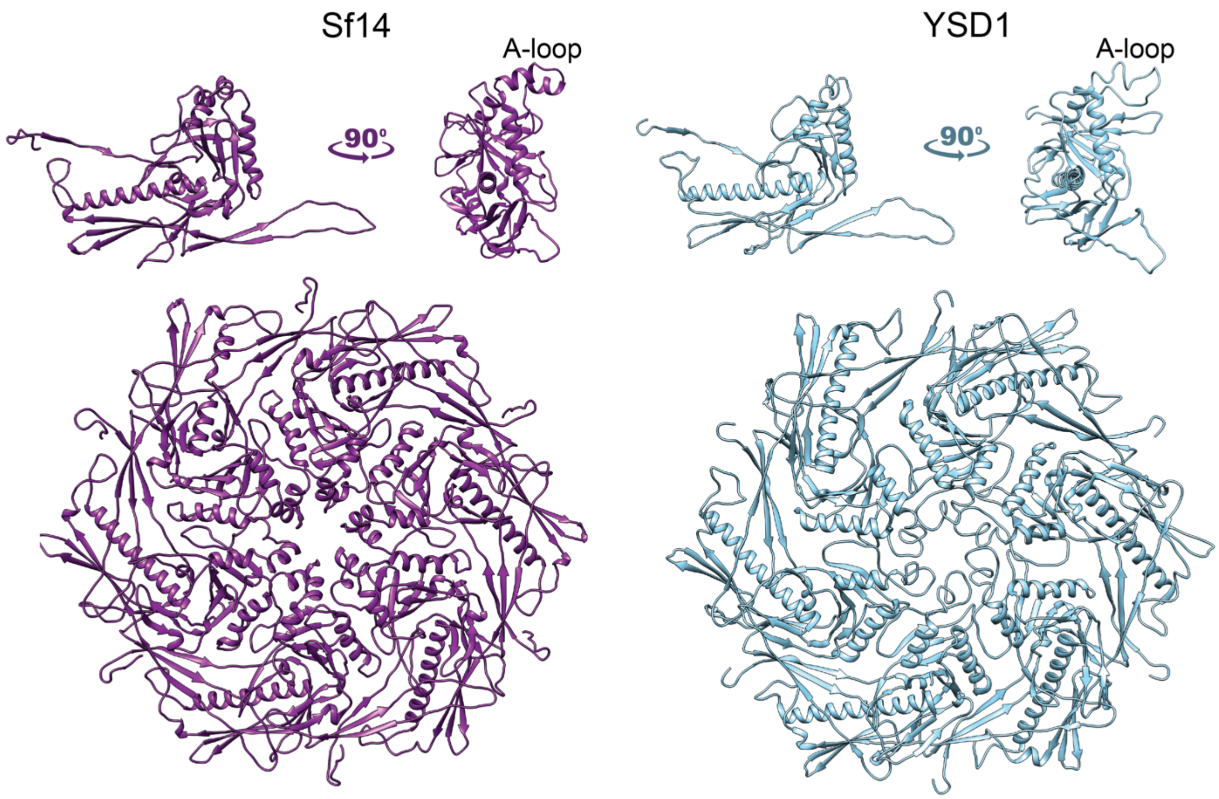

Supplemental Figure S2. Monomeric and hexameric Sf14 gp34 (left) compared to its closest DALI match, the YSD1 capsid protein (right; PDB 6XGQ). The A-loop, which is found at the center of each hexamer, is labeled on the monomer.

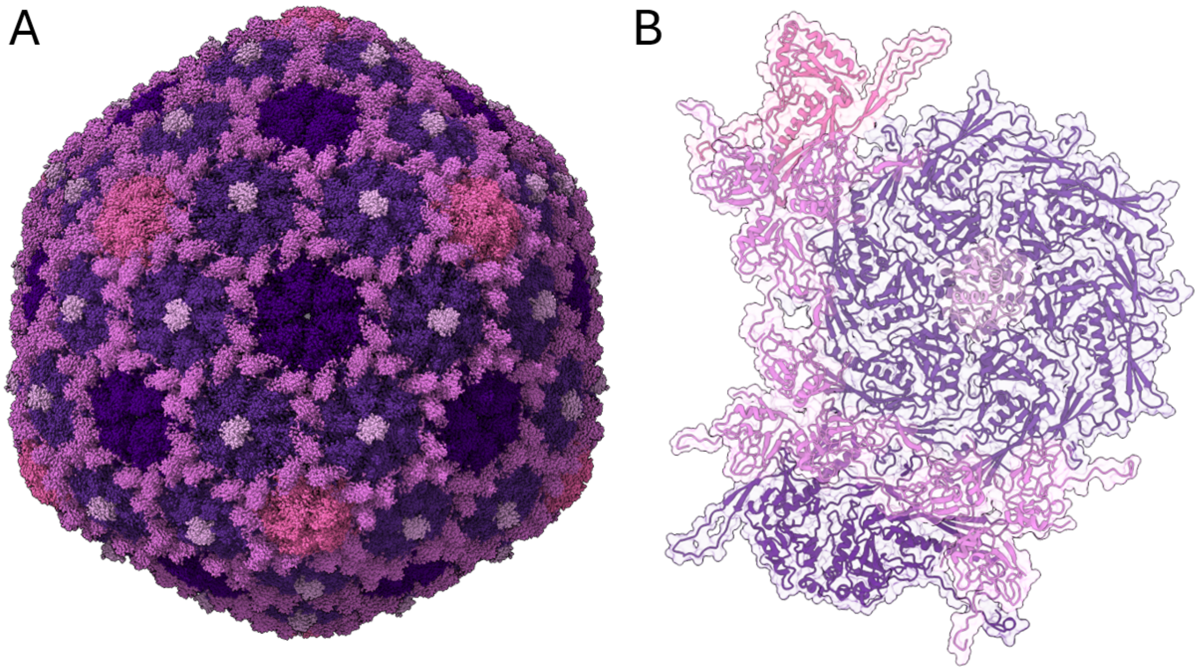

Supplemental Figure S3: A) The model of the Sf14 empty capsid and B) asymmetric subunit. This was reconstructed from particles without DNA as shown in Supplemental Figure S1A.

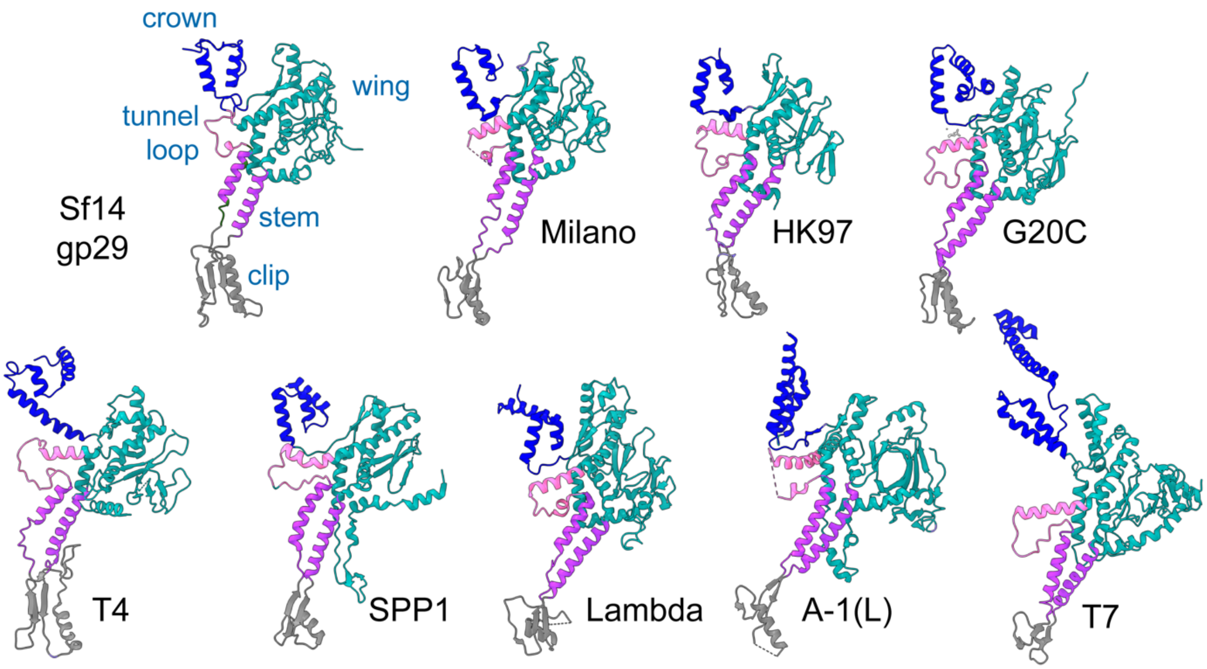

Supplemental Figure S4. Portal protein structures of Sf14 gp29 and its homologs Milano (8FWB), HK97 (1OHG), G20C (4ZJN), T4 (3JA7), SPP1 (2JES), lambda (8XOW), A-1(L) (8TS6), and T7 (7EY8). The Sf14 gp29 portal structure shown here was experimentally determined.

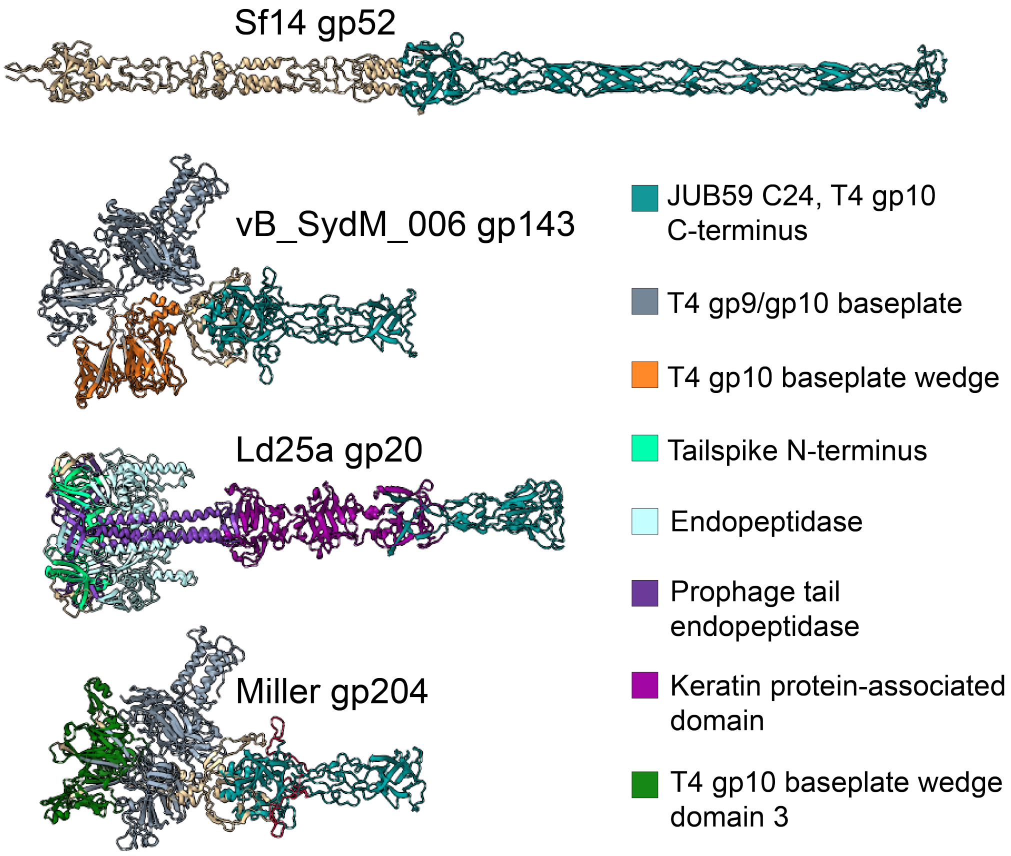

Supplemental Figure S5. The shorter tail fiber gp52 from *Shigella* phage domain architecture compared to: *Shigella* phage vB_SdyM_006 baseplate wedge subunit and tail pin gp143, *Lactobacillus* phage Ld25A putative anti-receptor gp20, and *Citrobacter* phage Miller baseplate wedge subunit and tail pin gp204. Regions are colored according to domain identity, as indicated on the right. All structures were determined computationally via AlphaFold.

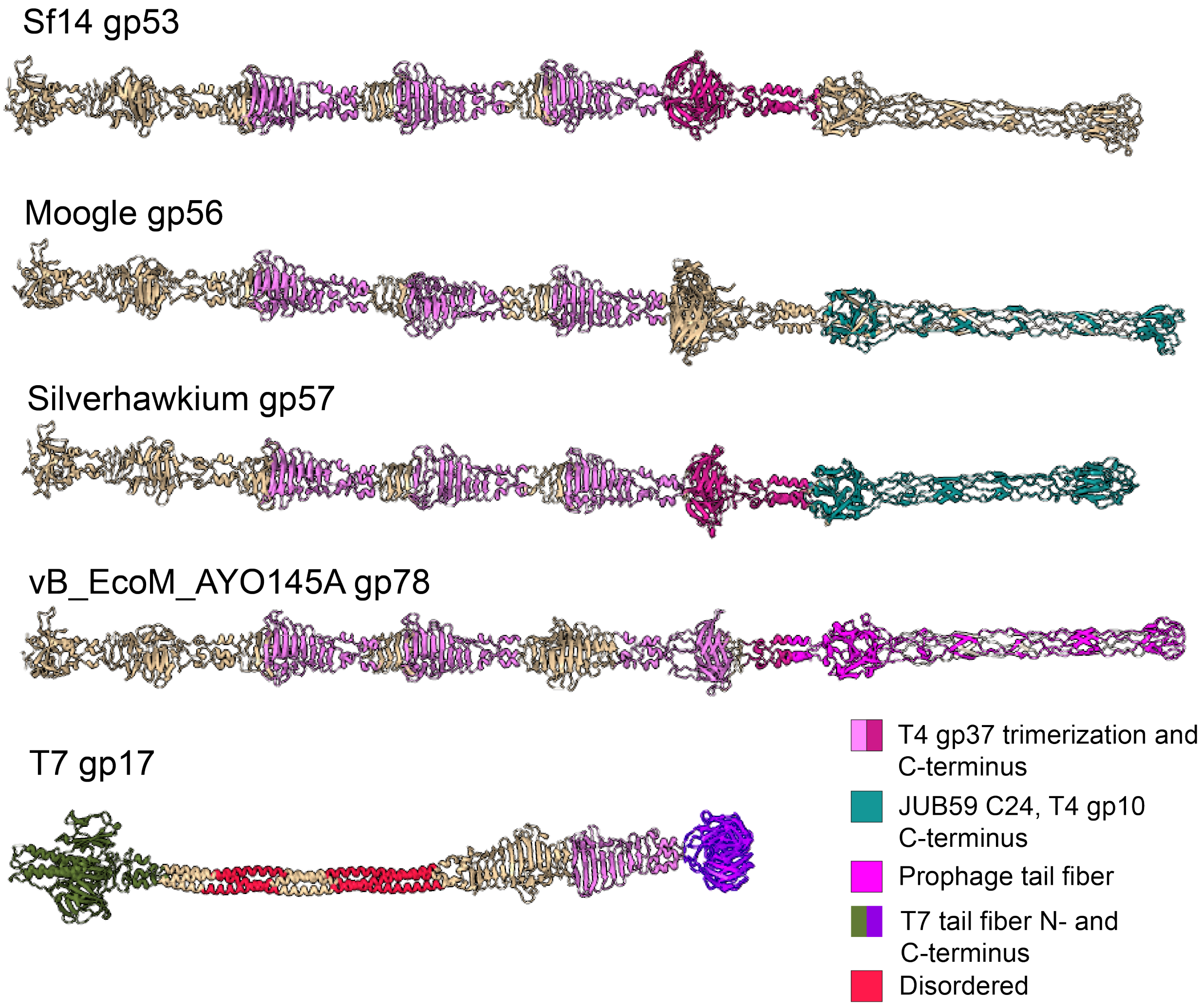

Supplemental Figure S6. Domain architecture comparison of the *Shigella* phage Sf14 longest tail fiber gp53 with other phages tail fibers: *Citrobacter* phage Moogle gp56, *Shigella* phage Silverhawkium gp57, *Escherichia* phage vB_EcoM_AYO145A gp78, and *Escherichia* phage T7 gp17. Regions are colored according to domain identity, as indicated on the lower right. All structures were determined computationally via AlphaFold for consistency.

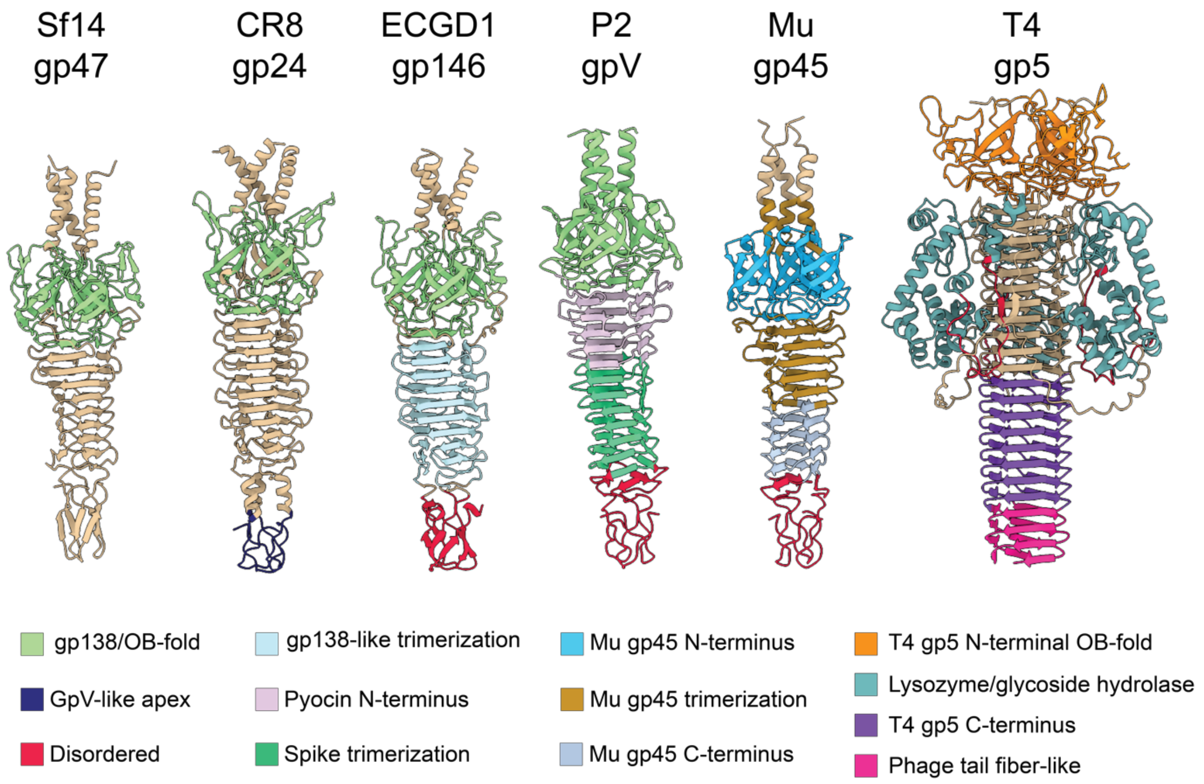

Supplemental Figure S7. Central tail needle comparison that contains Sf14 gp47 and proteins with similar domain architectures: *Cronobacter* phage CR8 gp24, *Enterobacteria* phage ECGD1 gp146, *Escherichia* phage P2 gene V, *Escherichia* phage Mu gp45, and *Escherichia* phage T4 gene 5. Regions are colored according to domain identity, as indicated below the structures. All structures were determined computationally via AlphaFold for consistency.

Supplemental Alignment

```
        M  I  K  A  K  T  Y  P  D  F  K  E  F  V  K  G  F  I  A  N
CHB7_gp95       ATGATTAAGGCAAAAACATATCCAGACTTCAAAGAGTTCGTTAAAGGCTTTATTGCGAAC 60
Sf14_gp20       ATGATTAAAGCAAAGACATATCCAGATTTTAAAGAGTTCGTTAAGGATTTTGTAGCGAAT 60
                 M  I  K  A  K  T  Y  P  D  F  K  E  F  V  K  D  F  V  A  N

                 V  K  A  G  K  R  Y  D  F  R  T  Y  Q  E  A  I  L  P  L  T
CHB7_gp95       GTTAAAGCTGGTAAAAGATATGACTTCAGAACATATCAAGAAGCAATTCTCCCACTGACT 120
Sf14_gp20       GTTAAAGCTGGCAAAAGATACGACTTCAGAAAGTATCAGGAGGCTGTTCTGCCTCTGACA 120
                 V  K  A  G  K  R  Y  D  F  R  K  Y  Q  E  A  V  L  P  L  T

                 Y  S  S  P  W  P  E  A  D  I  A  E  V  T  N  F  A  Y  E  P
CHB7_gp95       TACAGTTCGCCTTGGCCTGAAGCAGATATTGCAGAGGTGACCAACTTTGCTTATGAGCCA 180
Sf14_gp20       TATAGCTCACCGTGGCCTGAGTCTGATATTCCAGAAGTTACTGACTTCAATTACACACCA 180
                 Y  S  S  P  W  P  E  S  D  I  P  E  V  T  D  F  N  Y  T  P

                 A  Y  T  V  P  F  S  P  E  L  L  Y  S  V  G  A  Q  M  R  T
CHB7_gp95       GCATATACAGTCCCATTCAGTCCAGAACTTCTTTACAGTGTTGGTGCTCAAATGAGAACT 240
Sf14_gp20       GACTACACAGTTCCATTTAGTGAAGAACTTTTATATTCAGTTGGTGCTCAGATGAGGACT 240
                 D  Y  T  V  P  F  S  E  E  L  L  Y  S  V  G  A  Q  M  R  T

                 A  D  F  F  M  D  L  Q  Y  A  I  I  N  G  K  D  V  D  T  V
CHB7_gp95       GCTGATTTCTTCATGGATTTGCAGTATGCAATCATCAACGGTAAAGATGTTGATACGGTT 300
Sf14_gp20       GCTGACTTCTTCATGGACTTGCAGTATGCAATCATCAATGGGAAGGATGTTGATACAGTT 300
                 A  D  F  F  M  D  L  Q  Y  A  I  I  N  G  K  D  V  D  T  V

                 Y  C  E  W  L  A  R  V  K  P  F  S  M  L  N  A  K  L  K  D
CHB7_gp95       TACTGTGAATGGCTTGCAAGAGTTAAACCTTTCTCAATGCTGAACGCAAAACTGAAAGAT 360
Sf14_gp20       TATTGTGAATGGCTTGCAAGAGTGAAACCCTTCTCGATGCTGAATGCAAAACTTAAAGAC 360
                 Y  C  E  W  L  A  R  V  K  P  F  S  M  L  N  A  K  L  K  D

                 S  V  V  P  P  V  I  T  T  Q  P  T  N  Q  T  V  N  E  G  G
CHB7_gp95       TCTGTAGTTCCTCCAGTAATCACTACACAGCCAACTAACCAGACTGTTAATGAAGGTGGT 420
Sf14_gp20       TCTGCACAACCACCTGTAATCACCACACAACCAACTGGTGGCGCAGTTAATGAAGGTTCA 420
                 S  A  Q  P  P  V  I  T  T  Q  P  T  G  G  A  V  N  E  G  S
```

```
                 T  L  T  L  S  V  V  A  T  G  A  T  G  Y  Q  W  K  K  G  A 
CHB7_gp95       ACGTTGACTCTGAGTGTTGTTGCAACTGGTGCTACAGGTTATCAATGGAAGAAAGGTGCA 480
Sf14_gp20       GCAATTAATCTCAGCATTGTGGCAACAAATGCAACAAGCTACCAGTGGAAGAAGGGTAGT 480
                 A  I  N  L  S  I  V  A  T  N  A  T  S  Y  Q  W  K  K  G  S 

                 S  N  I  S  G  A  T  S  A  T  Y  T  K  T  N  V  V  P  A  D 
CHB7_gp95       AGTAACATCTCCGGTGCAACGTCTGCAACTTACACAAAAACAAACGTAGTCCCTGCTGAT 540
Sf14_gp20       TCAGATATCTCAGGTGCAACCTCAGCTACGTATACTAAGGCTGGCGCTGTACCAGCAGAT 540
                 S  D  I  S  G  A  T  S  A  T  Y  T  K  A  G  A  V  P  A  D 

                 A  G  S  Y  T  C  V  V  T  G  E  G  G  A  T  V  T  S  N  A 
CHB7_gp95       GCTGGTTCTTACACTTGTGTAGTAACTGGTGAAGGTGGTGCAACTGTAACGTCAAACGCT 600
Sf14_gp20       GCAGGAAGCTATACGTGCGTAGTAACAAATGATGTAGGCTC---TACAACATCTGATGCA 597
                 A  G  S  Y  T  C  V  V  T  N  D  V  G  S     T  T  S  D  A 

                 A  T  V  T  V  N  A  L  P  V  I  T  T  Q  P  Q  N  V  E  I 
CHB7_gp95       GCAACTGTGACGGTGAATGCTTTACCAGTAATCACTACACAGCCACAAAACGTTGAAATC 660
Sf14_gp20       GCAGTTATTACAATCAACCCGCTTCCAGTAATCACCACACAACCAACTAGCAAAGCTGTT 657
                 A  V  I  T  I  N  P  L  P  V  I  T  T  Q  P  T  S  K  A  V 

                 T  E  G  D  T  L  E  L  S  I  V  A  T  G  A  T  G  Y  Q  W 
CHB7_gp95       ACTGAAGGTGACACTCTTGAGTTGAGCATTGTGGCTACTGGAGCAACTGGTTACCAGTGG 720
Sf14_gp20       AATGAAAGTTCCACACTAACACTAAGCGTGGTTGCAACTGGAGCGACAAGCTATCAGTGG 717
                 N  E  S  S  T  L  T  L  S  V  V  A  T  G  A  T  S  Y  Q  W 

                 K  K  G  A  D  N  I  S  G  A  T  S  A  T  Y  T  K  E  N  A 
CHB7_gp95       AAGAAAGGTGCTGACAACATCTCTGGAGCAACTTCAGCAACTTACACAAAAGAGAATGCA 780
Sf14_gp20       AAGAAGAATGGTACAAATATCTCAGGTGCAACATCTGCAACTTACTCAAAAGCAAATGCT 777
                 K  K  N  G  T  N  I  S  G  A  T  S  A  T  Y  S  K  A  N  A 

                 T  T  A  D  D  A  G  T  Y  T  C  V  V  T  G  A  G  G  S  V 
CHB7_gp95       ACAACTGCTGACGATGCCGGAACGTACACTTGCGTAGTTACTGGTGCAGGTGGTTCTGTA 840
Sf14_gp20       AAGACAACTGACGCAGG---AAGCTATACGTGTGTCGTAACAAATGCTGTAGGTTCTGTA 834
                 K  T  T  D  A  G     S  Y  T  C  V  V  T  N  A  V  G  S  V 
```

```
                 T  S  N  A  A  T  V  V  V  N  E  V  G  G  *
CHB7_gp95        ACGTCAAACGCAGCTACTGTTGTGGTTAACGAAGTAGGAGGTTAATAATGCAAC-TCTCT 899
Sf14_gp20        ACTTCAAATGCAGCAACTGTGACAATCAACCCACTTCCAGTCATTACGGTTCAGCCACAA 894
                 T  S  N  A  A  T  V  T  I  N  P  L  P  V  I  T  V  Q  P  Q

CHB7_gp95        GATAAGGGGCTTGCAGCAATTAAGTTTTTTGAAGGGCTTCGTCTAGAAGCCTATAGAGAC 959
Sf14_gp20        GACCAAGACCTAACAGTTGGTCAGACACTA---ACAATCAGTATCACTGCAACTGGTGCA 951
                D  Q  D  L  T  V  G  Q  T  L     T  I  S  I  T  A  T  G  A

CHB7_gp95        TCTGCTGGAATCCCAACAATCGGGTACGGTACAATCCGTATTGGTGGCAAGCCTGTTACG 1019
Sf14_gp20        ACTGGTTACCAGTGGAGAAA------AGGTAACAGCAATATCTCAGGTGCGACTTCTGCA 1005
                 T  G  Y  Q  W  R  K        G  N  S  N  I  S  G  A  T  S  A

CHB7_gp95        ATGGGTATGAAGATTACTGCTTCACAAGCTGAACAG------------TATCTGCTTGCA 1067
Sf14_gp20        ACATATACCAAAGCAAGTGTAACAACTGCTGATGACGGTAATTATGATTGCGTTGTAACA 1065
                T  Y  T  K  A  S  V  T  T  A  D  D  G  N  Y  D  C  V  V  T

CHB7_gp95        GATGTTGAAAGCTATGTTGGGGCGGTAAACAAAGCTATCAAGGTTCCAACCTCT       1119
Sf14_gp20        AACGCTGTAGGTTCTGTAACTTCTCACCAAGCAAAAGTTCAGGTAACTGCATGA       1119
                 N  A  V  G  S  V  T  S  H  Q  A  K  V  Q  V  T  A  *
```

Supplemental References

1 Hardy, J. M. *et al.* The architecture and stabilisation of flagellotropic tailed bacteriophages. *Nat Commun* **11**, 3748 (2020). https://doi.org:10.1038/s41467-020-17505-w

2 Leiman, P. G., Shneider, M. M., Mesyanzhinov, V. V. & Rossmann, M. G. Evolution of bacteriophage tails: Structure of T4 gene product 10. *J Mol Biol* **358**, 912-921 (2006). https://doi.org:10.1016/j.jmb.2006.02.058

3 Yap, M. L. *et al.* Role of bacteriophage T4 baseplate in regulating assembly and infection. *Proc Natl Acad Sci U S A* **113**, 2654-2659 (2016). https://doi.org:10.1073/pnas.1601654113

4 Kostyuchenko, V. A. *et al.* The structure of bacteriophage T4 gene product 9: the trigger for tail contraction. *Structure* **7**, 1213-1222 (1999). https://doi.org:10.1016/s0969-2126(00)80055-6

5 Vinga, I. *et al.* Role of bacteriophage SPP1 tail spike protein gp21 on host cell receptor binding and trigger of phage DNA ejection. *Mol Microbiol* **83**, 289-303 (2012). https://doi.org:10.1111/j.1365-2958.2011.07931.x

6 Hyman, P. & van Raaij, M. Bacteriophage T4 long tail fiber domains. *Biophys Rev* **10**, 463-471 (2018). https://doi.org:10.1007/s12551-017-0348-5

7 Garcia-Doval, C. & van Raaij, M. J. Structure of the receptor-binding carboxy-terminal domain of bacteriophage T7 tail fibers. *Proc Natl Acad Sci U S A* **109**, 9390-9395 (2012). https://doi.org:10.1073/pnas.1119719109

8 Hu, B., Margolin, W., Molineux, I. J. & Liu, J. Structural remodeling of bacteriophage T4 and host membranes during infection initiation. *Proc Natl Acad Sci U S A* **112**, E4919-4928 (2015). https://doi.org:10.1073/pnas.1501064112

9 Guan, J., Ibarra, D. & Zeng, L. The role of side tail fibers during the infection cycle of phage lambda. *Virology* **527**, 57-63 (2019). https://doi.org:10.1016/j.virol.2018.11.005

10 Hendrix, R. W. & Duda, R. L. Bacteriophage lambda PaPa: not the mother of all lambda phages. *Science* **258**, 1145-1148 (1992). https://doi.org:10.1126/science.1439823

11 Mori, Y. *et al.* Determination of the three-dimensional structure of bacteriophage Mu(-) tail fiber and its characterization. *Virology* **593**, 110017 (2024). https://doi.org:10.1016/j.virol.2024.110017

12 Browning, C., Shneider, M. M., Bowman, V. D., Schwarzer, D. & Leiman, P. G. Phage pierces the host cell membrane with the iron-loaded spike. *Structure* **20**, 326-339 (2012). https://doi.org:10.1016/j.str.2011.12.009

13 Filloux, A. The rise of the Type VI secretion system. *F1000Prime Rep* **5**, 52 (2013). https://doi.org:10.12703/P5-52

14 Taylor, N. M. *et al.* Structure of the T4 baseplate and its function in triggering sheath contraction. *Nature* **533**, 346-352 (2016). https://doi.org:10.1038/nature17971

15 Yamashita, E. *et al.* The host-binding domain of the P2 phage tail spike reveals a trimeric iron-binding structure. *Acta Crystallogr Sect F Struct Biol Cryst Commun* **67**, 837-841 (2011). https://doi.org:10.1107/S1744309111005999

16 Harada, K. *et al.* Crystal structure of the C-terminal domain of Mu phage central spike and functions of bound calcium ion. *Biochim Biophys Acta* **1834**, 284-291 (2013). https://doi.org:10.1016/j.bbapap.2012.08.015

17 Suzuki, H., Yamada, S., Toyama, Y. & Takeda, S. The C-terminal domain is sufficient for host-binding activity of the Mu phage tail-spike protein. *Biochim Biophys Acta* **1804**, 1738-1742 (2010). https://doi.org:10.1016/j.bbapap.2010.05.003

18 Henrissat, B. *et al.* Conserved catalytic machinery and the prediction of a common fold for several families of glycosyl hydrolases. *Proc Natl Acad Sci U S A* **92**, 7090-7094 (1995). https://doi.org:10.1073/pnas.92.15.7090

19 Nakagawa, H., Arisaka, F. & Ishii, S. Isolation and characterization of the bacteriophage T4 tail-associated lysozyme. *J Virol* **54**, 460-466 (1985). https://doi.org:10.1128/JVI.54.2.460-466.1985
